# Inferring the Impact of Regulatory Mechanisms that Underpin CD8+ T Cell Control of B16 Tumor Growth *In vivo* Using Mechanistic Models and Simulation

**DOI:** 10.3389/fphar.2016.00515

**Published:** 2017-01-04

**Authors:** David J. Klinke, Qing Wang

**Affiliations:** ^1^Department of Chemical and Biomedical Engineering and WVU Cancer Institute, West Virginia UniversityMorgantown, WV, USA; ^2^Department of Microbiology, Immunology, and Cell Biology, West Virginia UniversityMorgantown, WV, USA; ^3^Department of Computer Science, Mathematics and Engineering, Shepherd UniversityShepherdstown, WV, USA

**Keywords:** ordinary differential equations, Bayesian statistics, B16, immunotherapy, adoptive cell transfer, in vivo mouse model

## Abstract

A major barrier for broadening the efficacy of immunotherapies for cancer is identifying key mechanisms that limit the efficacy of tumor infiltrating lymphocytes. Yet, identifying these mechanisms using human samples and mouse models for cancer remains a challenge. While interactions between cancer and the immune system are dynamic and non-linear, identifying the relative roles that biological components play in regulating anti-tumor immunity commonly relies on human intuition alone, which can be limited by cognitive biases. To assist natural intuition, modeling and simulation play an emerging role in identifying therapeutic mechanisms. To illustrate the approach, we developed a multi-scale mechanistic model to describe the control of tumor growth by a primary response of CD8+ T cells against defined tumor antigens using the B16 C57Bl/6 mouse model for malignant melanoma. The mechanistic model was calibrated to data obtained following adenovirus-based immunization and validated to data obtained following adoptive transfer of transgenic CD8+ T cells. More importantly, we use simulation to test whether the postulated network topology, that is the modeled biological components and their associated interactions, is sufficient to capture the observed anti-tumor immune response. Given the available data, the simulation results also provided a statistical basis for quantifying the relative importance of different mechanisms that underpin CD8+ T cell control of B16F10 growth. By identifying conditions where the postulated network topology is incomplete, we illustrate how this approach can be used as part of an iterative design-build-test cycle to expand the predictive power of the model.

## 1. Introduction

Recent clinical successes illustrate the potential of immunotherapy to treat cancer (Topalian et al., 2015). Yet, malignant cells create a tissue niche by changing how cells communicate and by disrupting host immunity in non-intuitive ways that can be different among patients and between humans and pre-clinical models (Laland et al., 2014; Klinke, 2014b, 2016). Identifying the relative importance of specific mechanisms that can disrupt host immunity in a particular system from experimental data is challenging using human intuition alone. Modeling and simulation can play an important role to integrate knowledge related to the underlying biology and assist natural intuition in interpreting acquired data from humans and animal models (Klinke, 2015). However, a common critique of mathematical modeling is that model predictions are based on values of model parameters that are not known. Here, we illustrate a new approach enabled by improved computational power that can be used to test whether our existing knowledge of the key components of a biological system and their interactions, that is the network topology, are sufficient to explain the observed data irrespective of a lack of knowledge regarding the associated parameter values.

Enthusiasm associated with cancer immunotherapies is driven by the remarkable clinical successes associated with modulating immune checkpoints and adoptive cell transfer of T cells that recognize tumor-associated antigens (Hodi et al., 2010; Porter et al., 2011; Rosenberg et al., 2011; Robbins et al., 2011; Topalian et al., 2012). While these therapies provide a significant clinical benefit to a subset of patients, the emerging challenge is to broaden the clinical benefit of these therapies across the patient population (Topalian et al., 2015). As a distributed and adaptive system, the ability of the immune system to defend against a malignancy depends on the collective action of multiple cell types that are distributed spatially throughout the body and provide information that changes with time and spatial context (Chen and Mellman, 2013; Spitzer et al., 2015). While there are multiple points where this process can become interrupted, these new immunotherapies jumpstart this process by increasing the number of CD8+ T cells that can infiltrate tumors (Herbst et al., 2014; Tumeh et al., 2014). Improving efficacy of immunotherapies then depends on improving the efficacy of CD8+ T cells that infiltrate the tumor microenvironment to recognize and kill malignant cells.

The tumor microenvironment is a complex environment where multiple cell types contribute to overall tumor growth. Identifying the roles that cells play in tissue dynamics, especially in the case of anti-tumor immune response, presents a challenge for human intuition alone. Models, either experimental or mathematical, aid in thinking clearly about how tumors disrupt host immunity by recreating this complicated cellular cross-talk in a system that can be manipulated. Transplantable mouse models are commonly used experimental models for testing whether a particular component plays a causal role in modulating anti-tumor immunity (Dranoff, 2012). While *in vivo* mouse models are considered the gold standard for testing mechanistic hypotheses, limited observability of a complicated dynamic, non-linear system can lead to non-intuitive results or limited translational relevance (Wen et al., 2012). Alternatively, math models aid in testing whether a mechanistic explanation is consistent with observed data by encoding prior knowledge of key components of a system and how these components are thought to interact (Shoda et al., 2010; Germain et al., 2011; Klinke, 2015). While the parameter values that quantify the relative importance of these interactions are largely unknown, computational tools can be used to select parameter values that are consistent with observed data and to test from a strong statistical viewpoint whether the postulated network is consistent with the observed data, that is *in silico* model-based inference (Klinke, 2014a, 2015). The complexity of a mathematical model can then be progressively increased to incorporate more biological detail through iterative design-build-test cycles.

To illustrate *in silico* model-based inference in the context of cancer immunotherapy, we developed a multi-scale mechanistic model to describe the control of tumor growth by a primary response of CD8+ T cells against defined tumor antigens using the B16 mouse model for malignant melanoma (Ya et al., 2015). The mechanistic model was calibrated to data obtained following adenovirus-based immunization to the tumor rejection antigen dopachrome tautomerase antigen (DCT) and the glycoprotein gp100 (Bloom et al., 1997; Overwijk et al., 1998). We used simulation to test whether the postulated network topology, that is the modeled biological components and their associated interactions, was sufficient to capture the observed system. The resulting model was then validated to data obtained following adoptive transfer of transgenic CD8+ T cells that recognized antigens derived from gp100. As part of an iterative approach, the validated model and associated predictions suggest that increasing the number of tumor infiltrating CD8+ T cells was necessary but not sufficient for CD8+ T cell-mediated control of tumor growth and outgrowth of B16F10 tumors depended on a transient loss of MHC class I antigen presentation. While the functional defects in CD8+ T cells that occur upon localizing to the tumor microenvironment is established (e.g., McGray et al., 2014), these simulations highlight how the relationship between tumor and CD8+ T cells can abruptly change with time following tumor transplant. Uncontrolled dynamics can have important implications for interpreting experimental results and the translational relevance of these pre-clinical mouse models.

## 2. Materials and methods

### 2.1. Models and inference

A multi-scale mathematical model was constructed to represent both prior knowledge about elements of the cellular network and postulated dynamic relationships among the observed components of the biological system. These causal relationships among the modeled biological components were represented using a mass-action formalism and encoded using a set of ordinary differential equations. Geometrically, these causal relationships, that is the model topology, can generate an infinite family of curves that trace all possible dynamic trajectories of the system in network state space. Individual curves are defined by specific values of the model parameters and initial conditions. Once the topology of the model is specified, a subset of these curves is selected based on goodness-of-fit with the specified experimental data. Using this subset of curves and associated parameter values, the model can be used to describe the evolution in the cellular network as a function of time and to explore the implications of the assumed model structure. This process of determining whether the postulated model topology is consistent with the experimental data, given the uncertainty in the model parameters, is called in silico model-based inference (Klinke, 2009; Klinke et al., 2012; Klinke, 2014a). To focus on the computational approach, experimental data was obtained from previously published studies that describe using an adenovirus vaccine against melanoma antigens to control the growth of the B16 mouse melanoma model (Yang et al., 2006; McGray et al., 2012, 2014). In the following subsections, the model topology and in silico model-based inference of the model parameters will be discussed in more detail.

#### 2.1.1. Mathematical model of the anti-tumor immunity initiated by adenovirus vaccination

**Figure 1:**
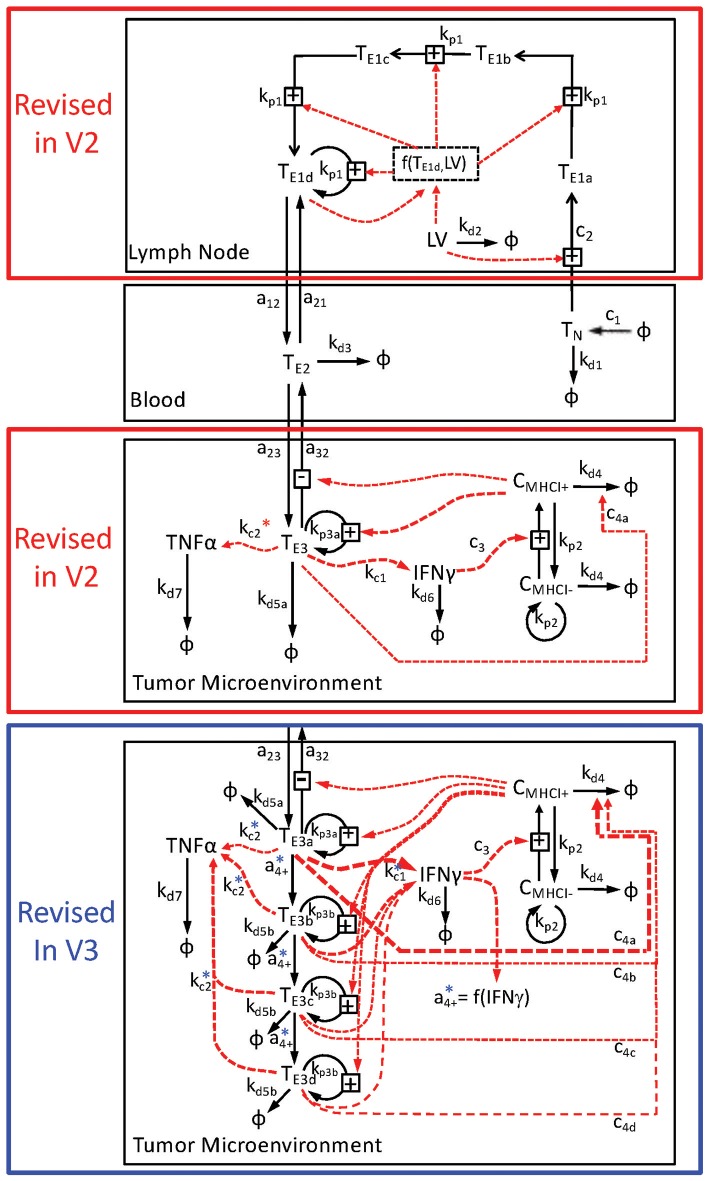
**Schematic diagram of multi-compartment model of adenovirus-induced control of B16F10 tumor growth**. The mathematical model is divided into three compartments: the lymph node (top), the blood (middle), and the tumor microenvironment (bottom two panels). The key state variables include CD8+ T cells that are denoted as T_*x*_ and cancer cells that are denoted by C_*x*_, where *x* indicates the specific subset. The immunization vector and cytokine states are represented by LV, TNFα, and IFNγ, respectively. Rate relationships associated with each compartment are contained within a black box associated with each compartment and indicated by arrows connecting two state variables. Transport of modeled state variables between compartments is represented by solid arrows that cross compartmental boundaries. The lymph node compartment was modeled differently in the V2 and V3 relative to the V1 model. In addition, the tumor microenvironment was modeled in two different ways. The V2 model, enclosed by the red box, contained just one state of effector CD8+ T cells in the tumor microenvironment, while the V3 model, enclosed by the blue box, contained four states of effector CD8+ T cells. Following from the detailed description presented in the Text, the conversion or transport of cells between modeled states are represented by the solid arrows. The dotted red arrows represent the influence of a given state variable on the conversion rate of another state variable. Each arrow is annotated by its corresponding rate constant. The rate constants associated with the production of TNFα in the tumor microenvironment (*k*_*c*2_) are also annotated with ^*^ to indicate that the actual rate relationships are more complicated than depicted in the diagram.

As summarized in Figure 1, two different modifications of a deterministic multi-scale mathematical model were formulated to describe the observed system and to integrate existing knowledge about CD8+ T cell-mediated killing of tumor cells following immunization against a tumor antigen using an adenovirus vector. To represent the spatial organization of anti-tumor immune response, three different compartments were created to discriminate between components observed in either the secondary lymph nodes, blood, or tumor microenvironment. The initial model is described previously (Wang et al., 2015) and referred to as V1. The two modified models are referred to as V2 and V3. The particular biological components included in the V2 and V3 models and a description of their corresponding rate equations are described in the next paragraphs. In brief, the V2 model modified the state variables in the lymph node compartment to account for an age-structured population and in the tumor microenvironment to account for changes in compartmental size. The V3 model modified the state variables in the tumor microenvironment.

- [*T*_*N*_]**: Naïve CD8**+ **T cells in the blood expressed in cells per mm**^3^. We assume that naïve CD8+ T cells that recognize the specific tumor antigen used in the adenovirus immunization are produced at a constant rate, *c*_1_, from thymus and die naturally at a rate *k*_*d*1_ · *T*_*N*_. To solve for the constant rate of production, we assumed that naïve CD8+ T cells are maintained at a constant level in the absence of the adenovirus, i.e., *c*_1_ = *k*_*d*1_ · *T*_*N*_(0). Upon immunization, naïve CD8+ T cells are recruited to the lymph node, activated by the adenovirus vector, and begin a proliferation and differentiation process that results in their ultimate conversion to effector CD8+ T cells. The third term in the rate equation represents the recruitment of naïve CD8+ T cells to the lymph node at a rate proportional to *T*_*N*_ and a saturable adenovirus-induced antigen (LV) term.
  (1) $$\begin{array}{l} \frac{dT_{N}}{dt}=c_{1}-k_{d1}\cdot T_{N}-c_{2}\cdot T_{N}\cdot \frac{\text{LV}}{\text{LV}+k_{g}} \end{array}$$
- [*T*_*E*1*a*−*d*_]**: Effector CD8**+ **T cells in the lymph node expressed in cells per mm**^3^. Newly activated naïve CD8+ T cells initiate a differentiation and polarization cell fate program to create effector CD8+ T cells. This process takes time and influences the functional characteristics that CD8+ T cells acquire during differentiation. To represent this age-structured process mathematically (see Klinke (2006)), we represent the differentiation of naïve into effector CD8+ T cells as a sequential series of intermediate cell states indicated by the species *T*_*E*1*a*_ through *T*_*E*1*d*_. The rate equation for the first cell state, *T*_*E*1*a*_, has two non-linear terms. The first term represents that recruitment of naïve CD8+ T cells to the lymph node, as similarly represented in Equation (1), and is multiplied by the ratio of the volume of the blood (*V*_*b*_) to volume of the lymph node (*V*_*ln*_) to account for changes in compartment size. The second non-linear term represents the rate of change associated with cell differentiation and proliferation, which also includes a saturable adenovirus-induced antigen (*LV*) term. Cell proliferation is also limited by immune checkpoints, where effector CD8+ T cells engage Cytotoxic T-lymphocyte antigen 4 (CTLA-4) that attenuates T cell receptor signaling (Sharma et al., 2011). This negative feedback on effector CD8+ T cell proliferation is represented by $\left(1-\frac{T_{E1d}^{2}}{k_{a}^{2}\;+\;T_{E1d}^{2}}\right)$. The presence of *T*_*E*1*d*_ in this term represents the up regulation of B7 homologue 1 (B7-H1), an early co-inhibitory membrane-expressed ligand that interacts with CTLA-4, on activated CD8+ T cells (Pulko et al., 2011). The other effector CD8+ T cell states also have similar non-linear terms. In the rate equation for most differentiated state in the lymph node, *T*_*E*1*d*_, the second term is positive as it represents the proliferation of this terminally differentiated state and the equation includes two additional terms that represent the trafficking of effector CD8+ T cells from the lymph node to the blood.
  (2) $$\begin{array}{l} \frac{dT_{E1a}}{dt}=c_{2}\cdot T_{N}\cdot \frac{V_{b}}{V_{ln}}\cdot \frac{\text{LV}}{\text{LV}+k_{g}}\\ -\;k_{p1}\cdot T_{E1a}\cdot \frac{\text{LV}}{\text{LV}+k_{g}}\;\{1-\frac{T_{E1d}^{2}}{k_{a}^{2}+T_{E1d}^{2}}\;\}, \end{array}$$
  (3) $$\begin{array}{l} \frac{dT_{E1b}}{dt}=2k_{p1}\cdot T_{E1a}\cdot \frac{\text{LV}}{\text{LV}+k_{g}}\;\{1-\frac{T_{E1d}^{2}}{k_{a}^{2}+T_{E1d}^{2}}\}\;\\ -\;k_{p1}\cdot T_{E1b}\cdot \frac{\text{LV}}{\text{LV}+k_{g}}\;\{1-\frac{T_{E1d}^{2}}{k_{a}^{2}+T_{E1d}^{2}}\;\}, \end{array}$$
  (4) $$\begin{array}{l} \frac{dT_{E1c}}{dt}=2k_{p1}\cdot T_{E1b}\cdot \frac{\text{LV}}{\text{LV}+k_{g}}\;\{1-\frac{T_{E1d}^{2}}{k_{a}^{2}+T_{E1d}^{2}}\}\;\\ -\;k_{p1}\cdot T_{E1c}\cdot \frac{\text{LV}}{\text{LV}+k_{g}}\;\{1-\frac{T_{E1d}^{2}}{k_{a}^{2}+T_{E1d}^{2}}\;\}, \end{array}$$
  (5) $$\begin{array}{l} \frac{dT_{E1d}}{dt}=2k_{p1}\cdot T_{E1c}\cdot \frac{\text{LV}}{\text{LV}+k_{g}}\;\{1-\frac{T_{E1d}^{2}}{k_{a}^{2}+T_{E1d}^{2}}\}\;\\ +\;k_{p1}\cdot T_{E1d}\cdot \frac{\text{LV}}{\text{LV}+k_{g}}\;\{1-\frac{T_{E1d}^{2}}{k_{a}^{2}+T_{E1d}^{2}}\;\}-\ldots \\ a_{12}\cdot T_{E1d}+a_{21}\cdot T_{E2}\cdot \frac{V_{b}}{V_{ln}}. \end{array}$$
- [LV]**: Adenovirus expression in the lymph node expressed in Relative Light Units (RLU) per mm**^3^**)**. The two adenovirus vectors used in this experiment encode for either the full length human dopachrome tautomerase (DCT) or the full length human melanoma Ag glycoprotein, gp100. The protein gp100 is a tissue-differentiation antigen that is expressed by both normal melanocytes and melanoma cells in humans and mice. Once the adenovirus is introduced on day 5, we assume an exponential decay model for LV with the rate constant of *k*_*d*2_, since the adenovirus used in the calibration experiments is replicate-defective:
  (6) $$\begin{array}{l} \frac{d\text{LV}}{dt}=\{\begin{array}{cc} 0,\; & \text{for }t\leq 5\text{ days}\\ -k_{d2}\;\cdot \text{ LV},\; & \text{for }t>5\text{ days} \end{array} \end{array}$$
  In addition, the concentration of LV was set equal to 10^5^ RLU per mm^3^ on day 5.
- **[***T*_*E*2_]**: Effector CD8**+ **T cells in the blood expressed in cells per mm**^3^. The model included one net source and two net sinks for effector CD8+ in the blood. The lymph node provides a net source of these cells, where the first and second terms represent the reversible trafficking of cells from the lymph node to the blood and back, respectively. The first term is multiplied by a volume ratio to account for the differences in compartment sizes between the blood and lymph node. The third term represents the natural death of effector CD8+ T cells in the blood, which is the first sink. The second sink is represented by the fourth and fifth terms that represent the reversible trafficking of cells from the blood into the tumor compartment and back, respectively. The migration rate of effector CD8+ T cells from the blood to the tumor is proportional to the concentration of cells in the blood. The trafficking of T cells from the tumor back to the blood is proportional to the fraction of tumor cells that do not express the antigen epitope recognized by the effector CD8+ T cells. The denominator in the fraction of tumor cells recognized by the CD8+ T corresponds to the total volume of the tumor ($V_{t}=\epsilon \;+\;C_{MHCI^{-}}\;+\;C_{MHCI^{+}}\;+\;V_{i}\cdot T_{E3x}$), where *V*_*i*_ is the volume of a single T cell (Abbas and Lichtman, 2003) and ϵ is small positive constant representing a small volume of tissue that excludes tumor and immune cells in the tumor compartment. The number of immune cells in the tumor compartment, *T*_*E*3*x*_, corresponds to either *T*_*E*3_ in the V2 model or the sum of all T cell states (*T*_*E*3*a*_ + *T*_*E*3*b*_ + *T*_*E*3*c*_ + *T*_*E*3*d*_) in the V3 model.
  (7) $$\begin{array}{l} \frac{dT_{E2}}{dt}=a_{12}\cdot T_{E1d}\cdot \frac{V_{ln}}{V_{b}}-a_{21}\cdot T_{E2}-k_{d3}\cdot T_{E2}-a_{23}\cdot T_{E2}\\ +\;a_{32}\cdot \frac{C_{MHCI^{-}}}{V_{t}}\cdot \frac{T_{E3a}}{V_{b}} \end{array}$$
- $\left[C_{MHCI^{-}}\right]$**: MHC class I negative tumor cells expressed in units of mm**^3^. Tumor cells can exist in one of two states: tumor cells that do not express the antigen epitope recognized by the effector CD8+ T cells and tumor cells that do express the epitope. The immunodominant peptide that is shared between human (the adenovirus antigen) and mouse (what CD8+ T cells recognize) DCT and binds to the relevant major histocompatibility complex (MHC) class I protein H-2K^*b*^ is DCT_180−188_, SVYDFFVWL (Parkhurst et al., 1998). For gp100, the immunodominant peptide that binds to H-2D^*b*^ is mgp100_25−33_, EGSRNQDWL (Overwijk et al., 1998). The first two terms represent the increase in tumor cells that are MHC class I negative due to cell proliferation. The first term represents the proliferation of MHC class I negative cells and the second term represents the increase in MHC class I negative cells due to proliferation of MHC class I positive cells that lose MHC class I expression due to cell division. In the absence of new protein synthesis and assuming symmetric cell division, copy numbers of proteins per cell is divided by 2 upon each cell division. While the copies of MHC class I is a continuous variable, discretizing the tumor cells into these two states based on MHC class I expression is a compact representation of this process. The third term represents the conversion of MHC class I negative to MHC class I positive tumor cells due to the dose-dependent action of Interferon γ. The final term represents the loss of tumor cells due to cell death independent of the cytotoxic action of effector CD8+ T cells. In contrast to the rest of the species in the model, the two tumor cell states are represented in terms of total volume rather than a concentration.
  (8) $$\begin{array}{l} \frac{dC_{MHCI^{-}}}{dt}=k_{p2}\cdot C_{MHCI^{-}}+2k_{p2}\cdot C_{MHCI^{+}}\\ -\;c_{3}\cdot \frac{\text{IFNG}}{k_{1}+\text{IFNG}}C_{MHCI^{-}}-k_{d4}\cdot C_{MHCI^{-}} \end{array}$$
- $\left[C_{MHCI^{+}}\right]$**: MHC class I positive tumor cells expressed in units of mm**^3^. The rate of change of tumor cells that express the immunodominant peptide recognized by the effector CD8+ T cells has five terms. The first term represents the conversion of MHC class I negative to MHC class I positive tumor cells due to the dose-dependent action of Interferon γ. The second term represents the loss due to conversion to MHC class I negative cells by cell proliferation. The third term represents the loss of tumor cells due to cell death independent of the cytotoxic action of effector CD8+ T cells. The last two terms represent the loss of tumor cells due to the cytotoxic action of effector CD8+ T cells, which is implemented differently in the V2 (Equation 9-V2) and V3 (Equation 9-V3) models. In the V3 model, effector CD8+ T cells exist in one of four states, *T*_*E*3*a*_ to *T*_*E*3*d*_. The product of $\frac{T_{E3x}}{V_{t}}\cdot \frac{C_{MHCI^{+}}}{V_{t}}$ can be conceptualized as the probability that effector CD8+ T cells and MHC class I positive tumor cells could interact in a given finite volume element. The rate coefficients, *c*_4*a*_ to *c*_4*d*_, represent the number of productive immune cell-tumor cell interactions that result in a tumor cytotoxic event per hour and was determined for each of the different treatment conditions. The subscripts *a* to *d* correspond to the cytotoxic activity of the effector CD8+ T cell states *T*_*E*3*a*_ to *T*_*E*3*d*_, respectively.
  (9-V2) $$\begin{array}{l} \frac{dC_{MHCI^{+}}}{dt}=c_{3}\cdot \frac{\text{IFNG}}{k_{1}+\text{IFNG}}\cdot C_{MHCI^{-}}-k_{p2}\cdot C_{MHCI^{+}}\\ -\;k_{d4}\cdot C_{MHCI^{+}}-c_{4a}\cdot \frac{T_{E3}}{V_{t}}\cdot \frac{C_{MHCI^{+}}}{V_{t}}\cdot V_{t} \end{array}$$
  (9-V3) $$\begin{array}{l} \frac{dC_{MHCI^{+}}}{dt}=c_{3}\cdot \frac{\text{IFNG}}{k_{1}+\text{IFNG}}\cdot C_{MHCI^{-}}-k_{p2}\cdot C_{MHCI^{+}}\\ \;-\;k_{d4}\cdot C_{MHCI^{+}}-(c_{4a}\cdot \frac{T_{E3a}}{V_{t}}+c_{4b}\cdot \frac{T_{E3b}}{V_{t}}+\;c_{4c}\cdot \frac{T_{E3c}}{V_{t}}\\ \;+c_{4d}\cdot \frac{T_{E3d}}{V_{t}})\cdot \frac{C_{MHCI^{+}}}{V_{t}}\cdot V_{t} \end{array}$$
- [*T*_*E*3_, *T*_*E*3*a*−*d*_]**: Effector CD8**+ **T cells in the tumor microenvironment expressed in number of cells**. In the V2 model, effector CD8+ T cells within the tumor are described by only one phenotype or state: *T*_*E*3_. In the V3 model, effector CD8+ T cells in the tumor are considered to be in one of four differentiation states: *T*_*E*3*a*_, *T*_*E*3*b*_, *T*_*E*3*c*_ and *T*_*E*3*d*_. These different T cell states are defined by differences in IFNG production, proliferation capacity, migration capacity, and cytotoxic killing ability. The equations for *T*_*E*3_ and *T*_*E*3*a*_ include four terms that are related to cell trafficking and cell fate, which are shown in Equations (10-V2,-V3), respectively. The two terms for cell trafficking include a term for the movement of effector CD8+ T cells from the blood (*T*_*E*2_) and their return to the blood compartments that is dependent on the fraction of total tumor cells that do not express the tumor antigen epitope (i.e., $C_{MHCI^{-}}$). The two additional terms relate to an increase in cell number due to cell proliferation, which is proportional to the presence of antigen-expressing tumor cells (i.e., $C_{MHCI^{+}}$) and a rate constant *k*_*p*3*a*_, and a decrease in cell number due to cell death with a rate parameter *k*_*d*5*a*_. The equation for *T*_*E*3*a*_ (Equation 10.1-V3) also includes an additional term to represent the conversion in cell phenotype from *T*_*E*3*a*_ to *T*_*E*3*b*_. Conversion in cell phenotype includes a constitutive rate with the rate parameter *a*_4_ and a rate that is proportional to the concentration of IFNG within the tumor microenvironment, which is parameterized by *k*_1_ and the maximum rate *a*_5_.
  (10-V2) $$\begin{array}{l} \frac{dT_{E3}}{dt}=a_{23}\cdot T_{E2}\cdot V_{b}-a_{32}\cdot T_{E3}\cdot \frac{C_{MHCI^{-}}}{V_{t}}\\ +k_{p3a}\cdot T_{E3}\cdot \frac{C_{MHCI^{+}}}{V_{t}}-k_{d5a}\cdot T_{E3} \end{array}$$
  (10.1-V3) $$\begin{array}{l} \frac{dT_{E3a}}{dt}=a_{23}\cdot T_{E2}\cdot V_{b}-a_{32}\cdot T_{E3}\cdot \frac{C_{MHCI^{-}}}{V_{t}}\\ +k_{p3a}\cdot T_{E3}\cdot \frac{C_{MHCI^{+}}}{V_{t}}-k_{d5a}\cdot T_{E3}-\ldots \\ \left(a_{4}+a_{5}\cdot \frac{IFNG}{k_{1}+IFNG}\right)\cdot T_{E3a} \end{array}$$
  In an initial analysis of the data, we found that we were unable to capture the trend associated with IFNG gene expression in the tumor microenvironment. As it is well established that tumors create an immunosuppressive microenvironment (Motz and Coukos, 2013), we decided to split the effector CD8+ T cell population into four states. The additional effector CD8+ T cell states in the tumor microenvironment; *T*_*E*3*b*_, *T*_*E*3*c*_, and *T*_*E*3*d*_; represent different phenotypes that exhibit progressively diminished cytotoxic effector functions, such as reduced IFNG production and cytotoxic killing ability. The equations for *T*_*E*3*b*_ to *T*_*E*3*d*_ include four terms that are related to cell fate. The first two terms represent progressive conversion in cell phenotype, such as conversion from *T*_*E*3*a*_ to *T*_*E*3*b*_ and *T*_*E*3*b*_ to *T*_*E*3*c*_, and depend on a constitutive rate constant (*a*4) or rate proportional to IFNG (*a*5). The last two terms represent cell proliferation at a rate proportional to the number of antigen-expressing tumor cells and the rate constant *k*_*p*3*b*_ and cell death with a rate parameter of *k*_*d*5*b*_.
  (10.2-V3) $$\begin{array}{l} \frac{dT_{E3b}}{dt}=\left(a_{4}+a_{5}\cdot \frac{IFNG}{k_{1}+IFNG}\right)\cdot T_{E3a}\\ -\left(a_{4}+a_{5}\cdot \frac{IFNG}{k_{1}+IFNG}\right)\cdot T_{E3b}+\ldots \\ k_{p3b}\cdot T_{E3b}\cdot \frac{C_{MHCI^{+}}}{V_{t}}-k_{d5b}\cdot T_{E3b} \end{array}$$
  (10.3-V3) $$\begin{array}{l} \frac{dT_{E3c}}{dt}=\left(a_{4}+a_{5}\cdot \frac{IFNG}{k_{1}+IFNG}\right)\cdot T_{E3b}\\ -\left(a_{4}+a_{5}\cdot \frac{IFNG}{k_{1}+IFNG}\right)\cdot T_{E3c}+\ldots \\ k_{p3b}\cdot T_{E3c}\cdot \frac{C_{MHCI^{+}}}{V_{t}}-k_{d5b}\cdot T_{E3c} \end{array}$$
  (10.4-V3) $$\begin{array}{l} \frac{dT_{E3d}}{dt}=\left(a_{4}+a_{5}\cdot \frac{IFNG}{k_{1}+IFNG}\right)\cdot T_{E3c}\\ +k_{p3b}\cdot T_{E3d}\cdot \frac{C_{MHCI^{+}}}{V_{t}}-k_{d5b}\cdot T_{E3d} \end{array}$$
- [**IFNG**]**: Interferon** γ **in the tumor microenvironment expressed in moles per mm**^3^. As the tumor mass constitutes a compartment that is changing in volume, the rate of change in the concentration of IFNG in the tumor microenvironment includes terms related to the rate of change in the number of IFNG molecules and terms related to changes in concentration due to the rate of change in the compartment size. These terms follow directly from the application of the product rule where a protein concentration (*C*) in a well-mixed compartment is a ratio of the number of molecules of a protein (*N*) divided by the volume of the compartment (*V*):
  (11) $$\begin{array}{l} \frac{dC}{dt}=\frac{d\left(N/V\right)}{dt}=\frac{1}{V}\cdot \frac{dN}{dt}-\frac{N}{V^{2}}\cdot \frac{dV}{dt}. \end{array}$$
  Using this general relationship, the specific equations used for the two cytokine concentrations, IFNG and TNFα, in the tumor microenvironment are shown in Equations (12, 13). We assume that IFNG is produced under these experimental conditions by active effector CD8+ T cells present in the tumor microenvironment and is consumed at a concentration-dependent rate. The total number of tumor cells is represented by *C*_*t*_.
  (12-V2) $$\begin{array}{l} \frac{d\text{IFNG}}{dt}=k_{c1}\cdot \frac{T_{E3}}{V_{t}}-k_{d6}\cdot \text{IFNG}+\frac{\text{IFNG}}{V_{t}}\cdot V_{i}\cdot k_{d5a}\cdot T_{E3}\\ +\;\frac{\text{IFNG}}{V_{t}}\cdot c_{4a}\cdot T_{E3}\cdot \frac{C_{MHCI^{+}}}{V_{t}}-\ldots \\ \;\frac{\text{IFNG}}{V_{t}}\cdot V_{i}\cdot a_{23}\cdot T_{E2}\cdot V_{b}\\ +\;\frac{\text{IFNG}}{V_{t}}\cdot V_{i}\cdot a_{32}\cdot T_{E3}\cdot \frac{C_{MHCI^{-}}}{V_{t}}-\ldots \\ \;\frac{\text{IFNG}}{V_{t}}\cdot V_{i}\cdot k_{p3a}\cdot T_{E3}\cdot \frac{C_{MHCI^{+}}}{V_{t}}\\ +\;\frac{\text{IFNG}}{V_{t}}\cdot \left(k_{d4}-k_{p2}\right)\cdot C_{t} \end{array}$$
  (12-V3) $$\begin{array}{l} \frac{d\text{IFNG}}{dt}=k_{c1}\cdot (\frac{T_{E3a}}{V_{t}}+\frac{T_{E3b}}{V_{t}}\cdot \frac{c_{4b}}{c_{4a}}+\frac{T_{E3c}}{V_{t}}\cdot \frac{c_{4c}}{c_{4a}}\\ \;+\frac{T_{E3d}}{V_{t}}\cdot \frac{c_{4d}}{c_{4a}})-k_{d6}\cdot \text{IFNG}+\ldots \\ \;\frac{\text{IFNG}}{V_{t}}\cdot V_{i}\cdot [k_{d5a}\cdot T_{E3a}\\ \;+\;k_{d5b}\cdot (T_{E3b}+T_{E3c}+T_{E3d})]+\ldots \\ \;\frac{\text{IFNG}}{V_{t}}\cdot (c_{4a}\cdot T_{E3a}+c_{4b}\cdot T_{E3b}+c_{4c}\cdot T_{E3c}\\ \;+\;c_{4d}\cdot T_{E3d})\cdot \frac{C_{MHCI^{+}}}{V_{t}}-\ldots \\ \;\frac{\text{IFNG}}{V_{t}}\cdot V_{i}\cdot a_{23}\cdot T_{E2}\cdot V_{b}\\ \;+\;\frac{\text{IFNG}}{V_{t}}\cdot V_{i}\cdot a_{32}\cdot T_{E3a}\cdot \frac{C_{MHCI^{-}}}{V_{t}}-\ldots \\ \;\frac{\text{IFNG}}{V_{t}}\cdot V_{i}\cdot [k_{p3a}\cdot T_{E3a}\\ \;+\;k_{p3b}\cdot (T_{E3b}+T_{E3c}+T_{E3d})]\cdot \frac{C_{MHCI^{+}}}{V_{t}}+\ldots \\ \;\frac{\text{IFNG}}{V_{t}}\cdot (k_{d4}-k_{p2})\cdot C_{t} \end{array}$$
- [**TNF**α]**: Tumor Necrosis Factor** α **in the tumor microenvironment expressed in moles per mm**^3^. Similar to the equations for the concentration of IFNG, the rate of change in TNFα in the tumor microenvironment includes terms that describe the production or consumption of TNFα molecules and terms that capture the change in concentration due to changes in compartmental size. The TNFα is produced by effector CD8+ T cells at a constant rate and this rate is increased in an autocrine positive feedback loop. TNFα present in the tumor microenvironment is eliminated with a concentration-dependent rate.
  (13-V2) $$\begin{array}{l} \frac{d\text{TNF}\alpha }{dt}=k_{c2}\cdot \frac{\text{TNF}\alpha }{k_{2}+{\text{TNF}}_{\alpha }}\cdot \frac{T_{E3}}{V_{t}}+k_{c3}\cdot \frac{T_{E3}}{V_{t}}-k_{d7}\cdot \text{TNF}\alpha \\ +\;\frac{\text{TNF}\alpha }{V_{t}}\cdot V_{i}\cdot k_{d5a}\cdot T_{E3}+\ldots \\ \;\frac{\text{TNF}\alpha }{V_{t}}\cdot c_{4a}\cdot T_{E3}\cdot \frac{C_{MHCI^{+}}}{V_{t}}-\ldots \\ \;\frac{\text{TNF}\alpha }{V_{t}}\cdot V_{i}\cdot a_{23}\cdot T_{E2}\cdot V_{b}\\ +\;\frac{\text{TNF}\alpha }{V_{t}}\cdot V_{i}\cdot a_{32}\cdot T_{E3}\cdot \frac{C_{MHCI^{-}}}{V_{t}}-\ldots \\ \;\frac{\text{TNF}\alpha }{V_{t}}\cdot V_{i}\cdot k_{p3a}\cdot T_{E3}\cdot \frac{C_{MHCI^{+}}}{V_{t}}\\ \;+\frac{\text{TNF}\alpha }{V_{t}}\cdot \left(k_{d4}-k_{p2}\right)\cdot C_{t} \end{array}$$
  (13-V3) $$\begin{array}{l} \frac{d\text{TNF}\alpha }{dt}=k_{c2}\cdot \frac{\text{TNF}\alpha }{k_{2}+{\text{TNF}}_{\alpha }}\cdot (\frac{T_{E3a}}{V_{t}}+\frac{T_{E3b}}{V_{t}}+\frac{T_{E3c}}{V_{t}}\\ \;+\frac{T_{E3d}}{V_{t}})+\ldots \\ \;k_{c3}\cdot (\frac{T_{E3a}}{V_{t}}+\frac{T_{E3b}}{V_{t}}+\frac{T_{E3c}}{V_{t}}+\frac{T_{E3d}}{V_{t}})\\ \;-k_{d7}\cdot \text{TNF}\alpha +\ldots \\ \;\frac{\text{TNF}\alpha }{V_{t}}\cdot V_{i}\cdot [k_{d5a}\cdot T_{E3a}\\ \;+\;k_{d5b}\cdot (T_{E3b}+T_{E3c}+T_{E3d})]+\ldots \\ \;\frac{\text{TNF}\alpha }{V_{t}}\cdot (c_{4a}\cdot T_{E3a}+c_{4b}\cdot T_{E3b}\\ \;+\;c_{4c}\cdot T_{E3c}+c_{4d}\cdot T_{E3d})\cdot \frac{C_{MHCI^{+}}}{V_{t}}-\ldots \\ \;\frac{\text{TNF}\alpha }{V_{t}}\cdot V_{i}\cdot a_{23}\cdot T_{E2}\cdot V_{b}\\ \;+\;\frac{\text{TNF}\alpha }{V_{t}}\cdot V_{i}\cdot a_{32}\cdot T_{E3a}\cdot \frac{C_{MHCI^{-}}}{V_{t}}-\ldots \\ \;\frac{\text{TNF}\alpha }{V_{t}}\cdot V_{i}\cdot [k_{p3a}\cdot T_{E3a}\\ \;+\;k_{p3b}\cdot (T_{E3b}+T_{E3c}+T_{E3d})]\cdot \frac{C_{MHCI^{+}}}{V_{t}}+\ldots \\ \;\frac{\text{TNF}\alpha }{V_{t}}\cdot (k_{d4}-k_{p2})\cdot C_{t} \end{array}$$

The model equations were encoded and evaluated in MatLab R2013a (The MathWorks, Natick, MA). As the experimental measurements do not directly correspond to individual molecular species in the mathematical model, the simulated concentrations of the species in the model were combined to represent the experimental measurements. To make a comparison with the experimental RT-PCR data, the values for TCR gene expression was based on the total concentration of effector CD8+ T cells in the tumor microenvironment and the values of IFNG and TNFα gene expression were based on the simulated rate of protein production relative to the volume of the compartment, as described by:

(9) $$\begin{array}{l} \text{TCR}\alpha \text{ mRNA}=BG_{1}+\beta_{1}\cdot \left(T_{E3a}+T_{E3b}+T_{E3c}+T_{E3d}\right)/V_{t}\; \end{array}$$

(10) $$\begin{array}{l} \text{IFNG mRNA}=BG_{2}+\beta_{2}\cdot (T_{E3a}+T_{E3b}\cdot \frac{c_{4b}}{c_{4a}}+T_{E3c}\cdot \frac{c_{4c}}{c_{4a}}\\ +\;T_{E3d}\cdot \frac{c_{4d}}{c_{4a}})/V_{t} \end{array}$$

(11) $$\begin{array}{l} \text{TNF}\alpha \text{ mRNA}=BG_{3}+\beta_{3}\cdot \left(k_{c2}\cdot \frac{TNF\alpha }{k_{2}+TNF\alpha }+k_{c3}\right)\\ \cdot \;\left(T_{E3a}+T_{E3b}+T_{E3c}+T_{E3d}\right)/V_{t}. \end{array}$$

Many biological assays, like qRT-PCR, measure relative changes in a measurand, like gene expression, above a non-specific background signal, which is represented by the parameters *BG*_1_, *BG*_2_, and *BG*_3_. In comparing the experimental gene expression measurements with model predictions, we used qRT-PCR measurements obtained from untreated mice to estimate a non-specific background signal. Initially, we considered the qRT-PCR measurements obtained from untreated mice using all time points to estimate this non-specific background signal, as the background signal should be constant. However, when comparing the time points, the measurements obtained at day 8 for all three genes were consistently higher than all of the other time points. We used a StudentÕs *t*-test to test the null hypothesis that values from day 8 were drawn from the same population as values from other days. The resulting *p*-value (<1e-6) suggests that it is unlikely that these samples come from the same population. Therefore, day 8 qRT-PCR values in untreated mice were considered outliers and removed from subsequent analyses.

Initial values for the tumor cells and naive CD8+ T cells were specified to measured values while the remaining biological states contained within the model were initially set to near zero (i.e., 2e-16). Summed squared error between experimental and simulated measurements was used to determine goodness-of-fit. Maximum expectation estimates for the calibrated parameters, shown in Tables S1, S2, were determined using an empirical Bayesian approach (Klinke, 2009), as described in the next section.

#### 2.1.2. *In silico* model-based inference of model predictions and parameters

An empirical Bayesian approach was used to estimate the uncertainty associated with the model predictions and parameters, given the available experimental data (Klinke, 2009). Briefly, we used an Adaptive Markov Chain Monte Carlo (AMCMC) algorithm to generate a sequence of states that represent samples drawn from the posterior distribution of the model predictions, given the uncertainty in the model parameters and the specific calibration data. This involves the calculation of the following integral:

(12) $$\begin{array}{l} P\left(Y_{M}|M\right)=\int_{-\infty }^{+\infty }\int_{-\infty }^{+\infty }P\left(Y_{M}|\Theta ,M\right)\cdot P\left(Y|\Theta ,M\right)\cdot P\left(\Theta |M\right)d\Theta dY. \end{array}$$

As the available data (*Y*) is a finite discrete set, integrating with respect to *Y* involves adding the comparisons between each model prediction (*Y*_*M*_) and the corresponding observation (*Y*) that are represented by the likelihood (*P*(*Y*|Θ, *M*)). Integration with respect to the parameters (Θ) is more difficult but can be accomplished using Markov Chain Monte Carlo (MCMC) methods enabled by improved computational power. MCMC methods collect parameter values that provide model predictions that are consistent with the observed data using a random walk in parameter space. Importantly, the collective Markov chain represents samples drawn from the integrand: *P*(*Y*_*M*_|*M*). A starting point in the parameter space for the Markov Chains was obtained via simulated annealing (Beers, 2007). To accelerate the equilibration of the model behavior, final values were captured after the simulated annealing step and used as initial values for the AMCMC simulations. Using an unbiased prior distribution, a learning period of 100,000 steps was used to establish the covariance of the proposal distribution. The proposed steps within parameter space were evaluated using a Metropolis-Hastings algorithm with a targeted acceptance fraction equal to 0.2.

The key question here is how long of a chain do you need to estimate *P*(*Y*_*M*_|*M*). Convergence criteria, like the Gelman-Rubin Potential Scale Reduction Factor (PSRF), are used to determine how long of a walk is necessary to provide consistent estimates of *P*(*Y*_*M*_|*M*) (Gelman and Rubin, 1992; Brooks and Gelman, 1998). The Gelman-Rubin potential scale reduction factor was applied to the model predictions to estimate the convergence of the Markov chain to the posterior distribution of the model predictions, which are shown in Figures S2, S5. Once the chains are converged, subsequent samples help fill out the contours of *P*(*Y*_*M*_|*M*) and the corresponding parameter values that provide these predictions. In addition, representative samples from the posterior distribution were obtained by retaining every 200th step of the cumulative Markov chain.

**Figure 2:**
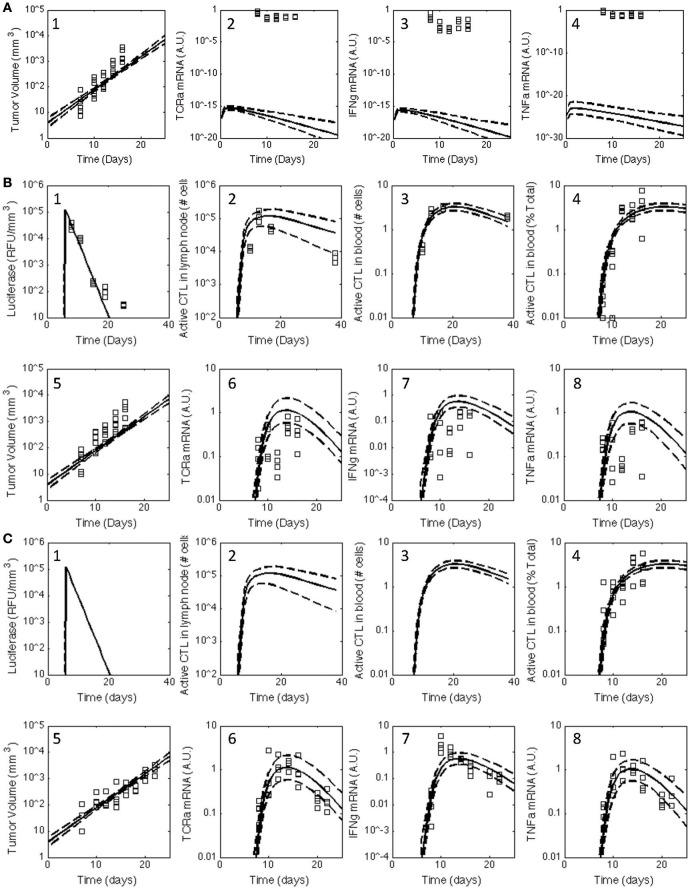
**Posterior distribution in the model predictions obtained for the V1 model compared against the observed data for all three treatment groups**. The experimental data (squares) acquired for untreated C57Bl/6 mice **(A)** and mice immunized with rHuAd5-hgp100 **(B)** and rHuAd5-hDCT **(C)** adenovirus expression vector on day 5 following subcutaneous implantation of 1 × 10^6^ B16F10 tumor cells were compared to the posterior distributions in the model predictions obtained using the V1 model. The comparisons between experimental measures of the anti-tumor immune response and corresponding simulated values are shown separately in subpanels. Experimental observations include antigen expression (luciferase expression: **B-1,C-1**), activated CD8+ T cells in lymph node **(B-2,C-2)**, activated CD8+ T cells in blood (percent of total CD8+ T cells: **B-3,B-4,C-3,C-4**), tumor volume **(A-1,B-5,C-5)**, TCRa mRNA expression in tumor **(A-2,B-6,C-6)**, IFNG mRNA expression in tumor **(A-3,B-7,C-7)**, and TNFα mRNA expression in tumor **(A-4,B-8,C-8)**. Using an empirical Bayesian approach, the uncertainty in the model predictions are represented by a solid line, which represents the median response, and dashed lines that enclose the 95% credible interval. Luciferase, active CD8+ T cells in lymph node, and activated CD8+ T cells in blood are not shown for untreated C57Bl/6 mice as they are essentially zero.

**Figure 3:**
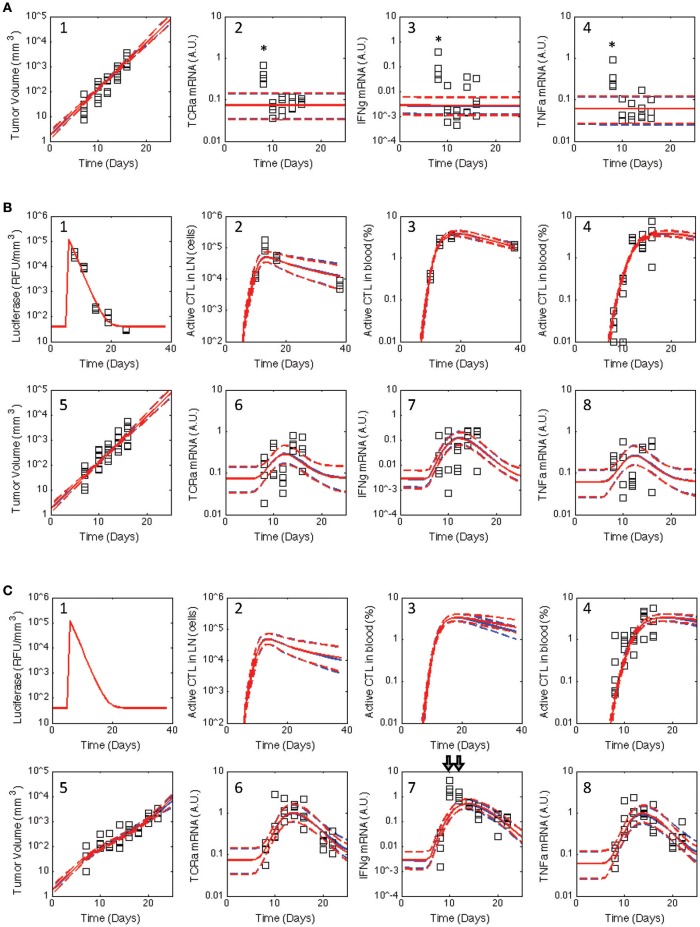
**Posterior distribution in the model predictions obtained for the V2 and V3 models compared against the observed data for all three treatment groups**. Similar to Figure 2, the experimental data (squares) acquired for untreated C57Bl/6 mice **(A)** and mice immunized with rHuAd5-hgp100 **(B)** and rHuAd5-hDCT **(C)** adenovirus expression vector on day 5 following B16F10 implantation were compared to the posterior distributions in the model predictions obtained using the V2 (red curves) and V3 (blue curves) models. Experimental observations shown in each subpanel include antigen expression (luciferase expression: **B-1,C-1**), activated CD8+ T cells in lymph node **(B-2,C-2)**, activated CD8+ T cells in blood (percent of total CD8+ T cells: **B-3,B-4,C-3,C-4**), tumor volume **(A-1,B-5,C-5)**, TCRa mRNA expression in tumor **(A-2,B-6,C-6)**, IFNG mRNA expression in tumor **(A-3,B-7,C-7)**, and TNFα mRNA expression in tumor **(A-4,B-8,C-8)**. The model predictions are represented by a solid line, which represents the median response, and dashed lines that enclose the 95% credible interval. As they were considered outliers, experimental values excluded from the analysis are indicated by ^*^. Luciferase, active CD8+ T cells in lymph node, and activated CD8+ T cells in blood are not shown for untreated C57Bl/6 mice as they are essentially zero.

Four parallel chains, each containing at least 1 × 10^6^ steps, were calibrated to the observed experimental data (see Figures 2, 3) and used to estimate the posterior distributions in the model predictions and parameters. The simulation of each chain took approximately 720 h on a single core of a 2.66 GHz Dual-Core Intel Xeon 64-bit processor with 8 GB RAM. A graphical summary of the Gelman-Rubin statistics was used as a diagnostic to determine convergence of the Markov chains to the posterior distribution in the model predictions (Figures S1, S4). An initial sequence of 3 × 10^5^ AMCMC steps was required for the four chains to converge. This initial sequence was used as the “burn-in” period. Traces for each of the parameters were used to estimate the degree of mixing among the four chains (see Figures S2, S5). The optimal parameter values were determined using the expectation maximum and are listed in Table S1. Pairwise scatter plots obtained from the four chains following the burn-in period were used to estimate the posterior identifiability of the model parameters (see Figures S3, S6). The scatter plots were colored based upon marginal posterior probability density obtained by kernel density estimation. A high value for the correlation coefficient suggests that the parameters were not independently identifiable given the calibration data. For example, the parameters associated with the rate of IFNG secretion (e.g., *k*_*c*1_) and the scaling constant for RT-PCR assay of IFNG gene expression (β_1_) were not practically identifiable, as they exhibited a correlation coefficient of nearly 1.0. The posterior distributions in the model predictions were obtained by marginalizing the model predictions over all of the parameter values from the Markov chains following the burn-in period. Despite the variation in the model parameters, the posterior predictions obtained from the converged segment of the Markov chains resulted in a narrow range of predictions (see Figure 3).

## 3. Results

### 3.1. A single effector CD8+ T cell state is unable to capture observed tumor response

Building upon a prior analysis of the B16 model for immunotherapy that generated the V1 model (Wang et al., 2015), we assembled a multi-scale model that represented key aspects of clonal expansion of CD8+ T cells in response to adenovirus vaccination against tumor antigens. The adenovirus vector induces both the transient expression of a defined tumor associated antigen and triggers innate immunity to initiate a primary adaptive immune response against this tumor antigen. As a primary adaptive immune response is organized spatially (Chen and Mellman, 2013), the model includes three compartments. First, a secondary lymph node compartment represents initial antigen presentation and activation of naive CD8+ T cells. Second, a blood compartment represents circulating naïve and effector CD8+ T cells in search of cognate tumor antigens. Third, a tumor microenvironment models infiltrating effector CD8+ T cells that can kill cells expressing the corresponding tumor antigen. The physiology represented by the model is shown schematically in Figure 1. We assumed that the size of the secondary lymph node and blood compartments remained fixed while the tumor microenvironment compartment changed in size due to changes in the number of B16F10 tumor cells. Motivated by inconsistencies between the V1 model and observed data that was not used in developing the V1 model (see Figure 2), parameters were incorporated to model immunizing against different tumor antigens. In addition, the representation of clonal expansion of antigen-specific CD8+ T cells in the lymph node was revised to represent an age-structured framework where naive CD8+ T cells must undergo a series of rounds of proliferation before they acquire the ability to emigrate from the lymph node as effector CD8+ T cells.

Cellular decision-making, like the acquisition of an effector phenotype by CD8+ T cells, is influenced by the intracellular abundance of proteins and can take multiple rounds of cell proliferation to change the cellular state (Marchingo et al., 2014; Kinjyo et al., 2015). Cellular decision-making and cell proliferation are, to some degree, linked and can be represented mathematically by the transition between discrete states. Within the cell, external signals are transmitted to the nucleus by modifying proteins post-translationally. The abundance of these modified proteins, in the absence of new external signals, is reduced by a factor of two with each cell division (Klinke et al., 2012). In the case of CD8+ T cells, antigen stimulation promotes the initial rounds of proliferation, which are guided by an intrinsic program of the cell. Subsequent rounds of proliferation are modulated by environmental signals, like IL-2, and correspond to downregulation of CD62L, migration to peripheral tissues, and acquisition of cytotoxic functionality (Jenkins et al., 2008; Hofer et al., 2012; Kinjyo et al., 2015). In addition to acquiring the ability to emigrate from the lymph node, they also upregulate co-inhibitory proteins, such as CTLA4 and B7-H1 (Pulko et al., 2011; Sharma et al., 2011), that aim to limit the clonal expansion of the T cell response. This process is represented in the model by a feedback term from effector CD8+ T cells (i.e., *T*_*E*1*d*_) that inhibits the rate of change associated with cell differentiation and proliferation in the lymph node (see Supplemental Text). These biological features were incorporated into a revised model, which is denoted as the V2 model.

The model was calibrated using the raw data summarized in a collection of three published studies that test whether immunization against tumor antigens could be used to control the growth of the B16F10 model of malignant melanoma, a widely used pre-clinical model for testing cancer immunotherapies (Ya et al., 2015). The first paper describes the dynamics of a primary CD8+ T cells response by immunizing with an adenovirus tumor-antigen delivery vehicle based on the recombinant human adenovirus serotype 5 (rHuAd5) vector (Yang et al., 2006). These data constrained the dynamics associated with adenovirus antigen expression and T cell dynamics in the lymph node and blood. Calibrating the effect of adenovirus vaccination on tumor growth was based on two studies (McGray et al., 2012, 2014). In these papers, the rHuAd5 vector is used to induce a CD8+ T cell response to human dopachrome tautomerase antigen (hDCT; vector: rHuAd5-hDCT) and to the glycoprotein gp100 (rHuAd5-hgp100). In the B16F10 model, DCT is a rejection antigen while gp100 is unable to be processed and presented by the Major Histocompatibility Complex class I pathway Leitch et al. (2004). As shown in (McGray et al., 2014), control of tumor growth in this model is through tumor-specific CD8+ T cells. Collectively, 448 data points were used to constrain 36 model parameters.

In contrast to Wang et al. where a single set of best-fit parameters were used, here *in silico* model-based inference was used to determine whether the postulated topology of this multi-scale model was consistent with the observed response of CD8+ T cell to adenovirus vaccination and concomitant changes in the tumor microenvironment under all three experimental conditions (untreated, rHuAd5-hgp100, and rHuAd5-hDCT). *In silico* model-based inference involves a random walk in parameter space to select parameter combinations that are consistent, within a certain probability, with the observed data (Klinke, 2009; Klinke et al., 2012; Klinke, 2014a). In theory, selecting appropriate parameter values was an over-determined problem because 448 data points were used to constrain 36 model parameters. However, in practice, selecting appropriate parameter values is a more difficult problem computationally and conceptually, as the information contained within each data point may not be unique and the model parameters may not have a unique impact on the model predictions (Klinke, 2014a). An adaptive Markov Chain Monte Carlo approach based on a Metropolis-Hastings algorithm, which is suited to these types of issues, was used to provide this random walk in parameter space; the computational details of the approach are described in the Supplemental Text. Converged segments from four independent Markov Chains were used to establish the posterior distributions in the model predictions and to establish an identifiable subset of model parameters (Figures S1–S3). Overall, the posterior distributions in the model predictions show good agreement with the observed experimental data (see red curves in Figure 3). In most cases, the dynamic trends were consistent between the model predictions and experimental observations despite the fact that the experimental variability was greater than the posterior distribution in model predictions. The small variability in the model predictions is consistent with the *in silico* model-based inference approach, where the experimental variability associated with each time point is averaged out by using a mechanistic model to integrate across the different time points.

To be more quantitative in comparing the two models, we used a Bayes Ratio (*B*_*ri*_) to quantify the strength of evidence that favors the revised model (*M*_*r*_) vs. initial model (*M*_*i*_) to capture the observed experimental data. The maximum value of the Bayes Ratio was obtained from the converged segments of the corresponding Markov Chains and calculated according to:

(13) $$\begin{array}{l} B_{ri}=\frac{Max\left(P\left(Y|\theta_{k},M_{r}\right)\right)}{Max\left(P\left(Y|\theta_{k},M_{i}\right)\right)}, \end{array}$$

where *P*(*Y*|θ_*k*_, *M*_*i*_) is the likelihood to observe data *Y*, given a set of parameter values θ_*k*_ and model *M*_*i*_. In turn, the likelihood is estimated by:

(14) $$\begin{array}{l} P\left(Y|\theta_{k},M\right)\propto {\left[\frac{1}{\sum_{j=1}^{N_{obs}}{\left(Y_{j}-Y_{Mj}\left(\theta_{k}\right)\right)}^{2}}\right]}^{\frac{N_{obs}}{2}}, \end{array}$$

where the denominator is the summed squared difference between the experimental observations, *Y*_*j*_, and the corresponding model predictions, *Y*_*Mj*_(θ_*k*_), and the exponent accounts for the number of experimental observations, *N*_*obs*_ (Klinke, 2014a). The Bayes Ratio was calculated separately for each observed state variable, as summarized in Table 1. Values for the *B*_*ri*_ between 1 and 3 are considered weak, between 3 and 20 are positive, between 20 to 150 are strong, and greater than 150 are very strong evidence favoring model *r* over model *i* (Klinke, 2014a). Collectively, the Bayes Ratios suggest strong to very strong evidence favoring the V2 over V1 model. Of all the values, simulations of the rHuAd5-hDCT-related data tend to have lower Bayes Ratios, which reflects the fact that the V1 model was developed primarily based on the rHuAd5-hDCT data. In contrast, the V2 model better captures the differences observed between these three experimental conditions, such as differences in tumor growth attributed to the presence of CD8+ T cells.

**Table 1:** **Bayes Ratios were used to quantify the evidence in support of successive revisions of the mathematical model**.

**Experimental**		**Bayes Ratio**	
**Group**	**Measure**	**V2 vs. V1**	**V3 vs. V2**
Untreated	Tumor volume	383.0	2.4
	TCRa mRNA	6.8E+31	1.0
	IFNG mRNA	5.9E+21	1.0
	TNFa mRNA	3.3E+32	1.0
rHuAd5-hgp100	Tumor volume	1.8E+11	1.0
	TCRa mRNA	74.6	1.5
	IFNG mRNA	68.3	0.9
	TNFa mRNA	2.5E+04	0.1
	Active CTL in blood	14.7	1.2
rHuAd5-hDCT	Tumor volume	1.3	2.0
	TCRa mRNA	17.7	1.4
	IFNG mRNA	0.01	2.4
	TNFa mRNA	287.6	1.7
	Active CTL in blood	1.0	0.2
	Tumor infiltrating T cells	1.0	1.0

*Using the simulation results for each model, the Bayes Ratio was calculated separately for each observed state variable according to Equation (13)*.

**Figure 4:**
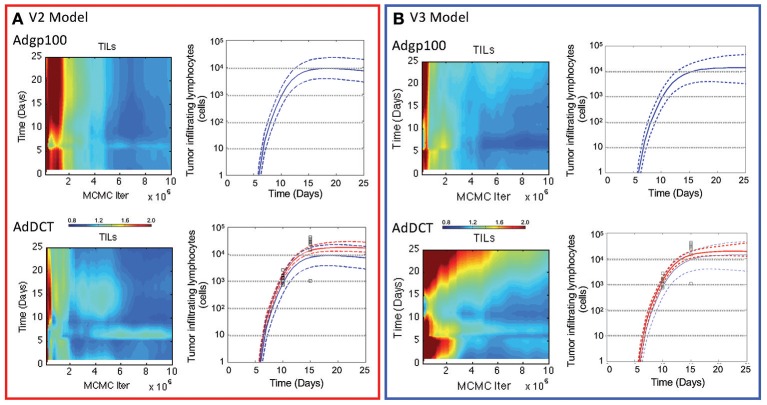
**Comparison of the number of tumor infiltrating lymphocytes in V2 vs. V3 models**. The number of CD8+ T lymphocytes infiltrating into implanted B16F10 tumors were simulated using the V2 **(A)** and V3 models **(B)**. Convergence of the model predictions as a function of the Markov Chain length were assessed using the Gelman-Rubin potential scale reduction factor (left subpanels). The posterior distribution in the model predictions obtained from the converged segments of the Markov Chains are represented by a solid line, which represents the median response, and dashed lines that enclose the 95% credible interval (right subpanels). Simulated values are compared against the number of tumor infiltrating lymphocytes (squares) that were quantified 10 and 15 days in tumors following implanting subcutaneously of 1 × 10^6^ B16F10 tumor cells and immunizing C57Bl/6 mice with DCT adenovirus on day 5 (bottom panels and red curves). The number of TILs were also simulated in mice immunized with rHuAd5-hgp100 (top panel and blue curves).

**Figure 5:**
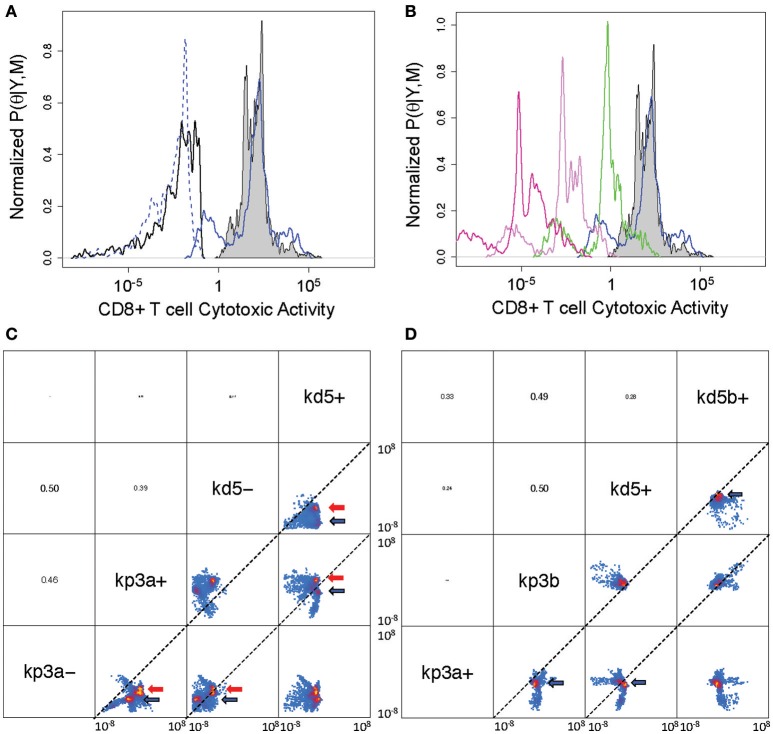
**Posterior distribution of parameters associated with cytotoxic killing ability and cell fate within the tumor microenvironment. (A)** Posterior density distributions of the parameter values associated with cytotoxic activity of CD8+ T cells, which corresponds to TE3 in the V2 model and TE3a in the V3 model. The parameter values, c4a for TE3 in the V2 model and c4a for TE3a in the V3 model, were determined separately based on rHuAd5-hgp100 and rHuAd5-hDCT vaccination data (c4 for rHuAd5-hgp100: solid black line, c4 for rHuAd5-hDCT: gray shaded, c4a for rHuAd5-hgp100: dotted blue line, c4a for rHuAd5-hDCT: solid blue line). **(B)** Posterior density distributions of the parameter values associated with the cytotoxic activity of CD8+ T cells following rHuAd5-hDCT vaccination in successive states of deactivation (TE3a: blue line, TE3b: green line, TE3c: indigo line, TE3d: purple line). The distribution in c4 for the V2 model is shown for comparison (gray shaded). **(C)** Pairwise scatter plots for the rate constants associated with cell proliferation (kp3a- and kp3a+) and cell death (kd5- and kd5+) of newly emigrating CD8+ T cells (TE3 in the V2 model and TE3a in the V3 model). A minus (−) and a plus (+) indicate whether the parameter values were determined using the rHuAd5-hgp100 or rHuAd5-hDCT data, respectively. Parameter values are shown for both the V2 and V3 revised models, where high probability regions are indicated by the red and blue arrows, respectively. The dotted lines indicate where the two parameter have equal values. **(D)** Pairwise scatter plots for the rate constants associated with cell proliferation (kp3a+ and kp3b) and cell death (kd5+ and kd5b+) of the different states of CD8+ T cells within the tumor. The parameters kp3a+ and kd5+ correspond to TE3a while kp3b and kd5b+ correspond to TE3b, TE3c, and TE3d. In **(C,D)**, parameter names are given on the diagonal. Above the diagonal are the pairwise correlation coefficients of the parameters, where the font size is proportional to the value of the correlation coefficient. Pairwise projections of the marginalized probability density in log_10_ space are given below the diagonal. Coloring is based upon the estimated 2-D posterior density distributions using kernel density estimation. The axes for the scatter plots each spans from 10^−8^ to 10^8^.

**Figure 6:**
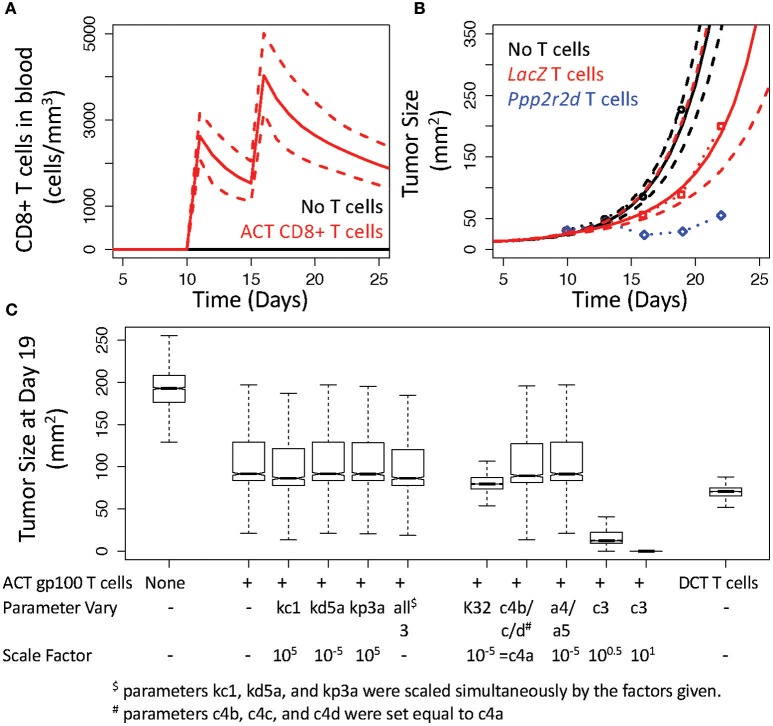
**V3 model predicts inhibition of B16 tumor growth by adoptive cell transfer of gp100 CD8+ T cells. (A)** Simulated response in the number of CD8+ T cells under baseline conditions (black) and following adoptive cell transfer (ACT) of CD8+ T cells that occurs 10 and 15 days after implanting B16F10 tumor cells. **(B)** Simulated growth in B16F10 tumors under baseline conditions (black curves) and following ACT of gp100 CD8+ T cells (red curves). Simulations were compared to similar experiments reported in (Zhou et al., 2014) where B16 tumor size was reported in mice without ACT of T cells (black circles) and in mice receiving ACT of pmel-1 CD8+ T cells that had been silenced for *LacZ* (red circles) or for *Ppp2r2d* (blue circles). In **(A,B)**, the most likely predictions are represented by the solid lines and the long-dashed lines enclose the corresponding 95% credible intervals. **(C)** Sensitivity analysis was performed by simulating tumor size at 19 days following changing the corresponding parameters of the V3 model. The box and whisker plots summarize the distribution in the model predictions simulated using the given change in a model parameter value and an ensemble of parameter values obtained from the converged segments of the Markov Chains.

**Figure 7:**
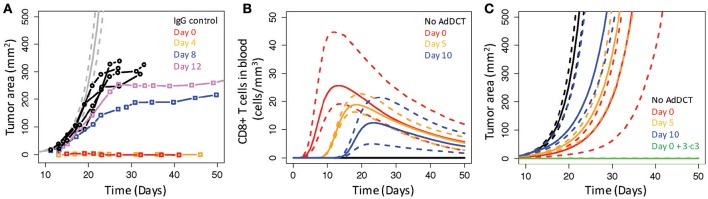
**Real B16 tumors incorporate time-dependent factors that limit CD8+ T cell control of tumor cell growth. (A)** Simulated growth of B16 tumors in untreated mice (gray curves) were compared against similar experiments reported in (van Elsas et al., 1999) where growth of B16 tumors was reported in mice treated with an IgG control (black circles) or with an anti-CTLA4 mAb and a vaccine comprised of irradiated B16 cells modified to express GM-CSF (BL6/GM). Administering the anti-CTLA4 plus the BL6/GM vaccine was varied from 0 (red squares), 4 (yellow squares), 8 (blue squares), and 12 (violet squares) days following tumor implantation. **(B,C)** Intradermal challenge by B16F10 cells was simulated under different conditions where mice were untreated (black curves) or immunized 0 (red curves), 5 (yellow curves), and 10 (blue curves) days with rHuAd5-hDCT following the intradermal challenge. Simulated immunization on day 0 (green curves) or on day 10 (not shown) was also combined with an increase in *c*3 by a factor of 3. Simulation results are shown for the number of CD8+ T cells in the blood **(B)** and tumor area **(C)**. The most likely predictions are represented by the solid lines and the long-dashed lines enclose the corresponding 95% credible intervals.

Of particular interest was whether the postulated topology of this multi-scale model can capture the dynamics associated with CD8+ T cell infiltration into the tumor microenvironment. Experimentally, the expression of genes associated with T cells (TCRa) and the secretion of cytokines related to their biological function (IFNG and TNFα) were measured in samples obtained from homogenized tumor samples. As a variety of other immune cells can elicit anti-tumor immunity, the adenovirus vector expressing hgp100 was used as a negative control as B16F10 cells have a defect in the MHC class I antigen processing machinery such that the immunodominant epitope from hgp100 is not processed and presented on the cell surface (Leitch et al., 2004). As there was no significant difference in tumor growth between untreated and hgp100-immunized animals, the contribution of other immune cells to control tumor growth was considered as negligible in this system. In addition, the observed response can be attributed to the response of CD8+ T cells to a tumor antigen, as tumor growth was inhibited in hDCT-immunized animals. In terms of characterizing CD8+ T cell infiltration, TCRa and TNFα mRNA exhibited symmetric peaks in expression that reached a maximum at day 14. In contrast, IFNG mRNA exhibited a different dynamic trend than either TCRa or TNFα gene expression. The profile of IFNG gene expression exhibited a skewed distribution that peaked at day 10 and declined thereafter. As illustrated by the inconsistency between the model predictions and observed data (see arrows in Figure 3C–7), a model that represents tumor-infiltrating lymphocytes as a single state does not appear to explain the observed data.

### 3.2. Representing tumor-infiltrating CD8+ T cells as a multi-state population better represents the observed tumor response

In the V1 and V2 models, we assumed that the phenotype of CD8+ T cells that enter the tumor in response to rHuAd5-hgp100 and to rHuAd5-hDCT are the same and remain constant in time, which implies that dynamic changes in IFNG should track changes in TCRa gene expression. The model predictions are consistent with the RT-PCR data obtained from rHuAd5-hgp100 treated mice. However, in rHuAd5-hDCT treated mice, there is a disconnect between IFNG and TCR gene expression that becomes apparent at days 10 and 12. Assuming that the disconnect between the predicted and observed dynamic change in IFNG gene expression is driven by an underlying biological process, we created a revised model (V3) that changed the representation of tumor-infiltrating lymphocytes from one state to four states. While our biological understanding should be used as a basis for modifying the model, what happens to CD8+ T cells once they enter the tumor microenvironment is somewhat unclear. What is known is that within a tissue hosting an ongoing type 1 immune response, CD8+ T cells are functionally heterogeneous (Halle et al., 2016). Within regressing tumors, CD8+ T cells proliferate (Tumeh et al., 2014) but also lose functionality compared to CD8+ T cells in the periphery (Grinshtein et al., 2009). Based on these observations, the model represents a hypothesis that this loss of functionality corresponds to a similar process as the acquisition of an effector phenotype but in reverse order.

Representing TILs as four related states corresponds to an age-structure, similar to the T cell states in the lymph node (see schematic diagram highlighted by blue box in Figure 1). The specific number of states, four, was a compromise between a one-state model and a model that represents cellular phenotype using an additional independent continuous variable and the discrete impact of cell division on cellular decision-making. The four states of tumor-infiltrating lymphocytes were defined to have a progressive decrease in IFNG gene expression and cytotoxic killing ability. As it unclear as to what specific mechanisms regulate the conversion of tumor-infiltrating lymphocytes among these different states, the rate of interconversion was parameterized by a constitutive rate constant and a rate constant that was proportional to the concentration of IFNG within the tumor microenvironment. While the specific biochemical details may be different in reality, the dependence on IFNG represents a negative feedback mechanism whereby an ongoing anti-tumor immune response inhibits the cell-mediated cytotoxic activity. Model-based inference was then used to determine whether the observed data support a constitutive rate for cellular deactivation vs. a negative feedback mechanism. Collectively, modifying the model to include an age-structure increased the number of free parameters from 36 to 41.

Similar to the V2 model, an adaptive Markov Chain Monte Carlo approach was used to obtain a posterior distribution in the predictions derived from the V3 model (see blue curves in Figure 3 and Figures S4–S6). Similarly, the posterior distributions in the model predictions show good qualitative agreement with the observed experimental data. We also compared predictions of the number of tumor infiltrating lymphocytes (Figure 4), which were qualitatively similar from the two models. A Markov Chain of 200,000 steps was required for the predictions to converge for the initial model. The V3 model also required about 200,000 steps for predictions that were constrained by data (10–15 days) but longer chains were required for predictions longer than 15 days to converge. Predictions after 17 days seemed to diverge between the two models where number of TILs predicted by the initial model peaked at 17 days, which mirrors the dynamics of the blood T cell population. The number of TILs predicted by the revised model reached a similar value at 17 days but then continued to increase at a slower rate through 25 days. For both V2 and V3 models, the posterior distribution in the number of TILs is broader than but enclose the rHuAd5-hDCT predictions, which is not surprising as there are no TILs data for the rHuAd5-hgp100 conditions. While predictions derived from both models match the number of TILs at 10 days, the models tend to predict a lower number of TILs at 15 days than observed on average.

In comparing V3 to V2 models, the Bayes Ratios were mostly between 1 and 3 suggesting weak but supportive evidence that V3 better represents the observed data over V2. The highest Bayes Ratio (2.4) was obtained in comparing the model predictions with the IFNG mRNA data observed following rHuAd5-hDCT immunization, as expected. For comparison, a Bayes Ratio of 540 is possible if the model was able to match the mean IFNG mRNA values on days 10 and 12. While alternative forms of the models were tested, an improved fit to the rHuAd5-hDCT data came at the expense of capturing the rHuAd5-hgp100 data. Assuming that these data reflect the underlying biology, these results imply that new CD8+ T cell immigrants to the tumor have a higher level of IFNG mRNA in rHuAd5-hDCT compared to rHuAd5-hgp100 immunized animals and that IFNG mRNA expression is regulated dynamically by cognate TCR interactions. This observation is at odds with the model, which assumes that immigrating CD8+ T cells have the same phenotype as CD8+ T cells in the circulation and that the phenotype of CD8+ T cells can be subsequently modified by the local microenvironment. Moreover, the phenotype of CD8+ T cells in the circulation should not depend on the specific antigen used in the vaccine. While failed killing correlates with cytokine hypersecretion (Jenkins et al., 2015), this phenomenon is unlikely to explain the early peak in IFNG as it should be linked in the model to the later CD8+ T cell states (i.e., *T*_*E*3*b*−*d*_) rather than new immigrants (i.e., *T*_*E*3*a*_) and a corresponding increase in TNFα mRNA with IFNG mRNA was not observed. Supported by the Bayes Ratios, the V3 model is an improvement. In addition, analyzing the data collectively identified underappreciated aspects of the dynamic CD8+ T cell response and suggests specific experimental conditions that may help in clarifying this process. We next examined the associated parameter distributions to gain insight into the underlying biological mechanisms represented in the corresponding models.

### 3.3. Deactivation of tumor infiltrating lymphocytes alters cell fate preference

Immunizing against tumor antigens aims to enhance the number of CD8+ T cells in the tumor microenvironment that recognize and kill tumor cells. Control of tumor growth depends on two factors, the ability of a CD8+ T cell to kill tumor cells and the number of CD8+ T cells present within the tumor microenvironment that recognize tumor antigens. As the distribution in parameter values is also influenced by the data, the V2 and V3 models were used to interpret the data to gain insight into these two factors by examining the distributions in parameters associated with these biological processes. In the following two paragraphs, we examine separately how the models parse the overall response into changes in cytotoxic activity vs. cell population dynamics among the different experimental conditions studied.

First, we consider the cytotoxic activity of CD8+ T cells within the tumor microenvironment predicted by the V2 and V3 models. In analyzing the data obtained following immunization using rHuAd5-hgp100, the posterior distributions in the parameters associated with CD8+ T cell killing are similar between the V2 and V3 models, where the median value for *c*4*a* in the V2 model was 0.0084 mm^6^ cells^−1^ day^−1^ and was 0.0038 mm^6^ cells^−1^ day^−1^ in the V3 model (see Figure 5A). In contrast to rHuAd5-hgp100, immunizing with rHuAd5-hDCT is more effective in enhancing the cytotoxic activity of CD8+ T cells as the killing parameter is at least a factor of 10,000 higher. As expected, newly immigrant CD8+ T cells in the V3 model are more effective in killing B16F10 cells than CD8+ T cells predicted by the V2 model (118.4 vs. 101.8). The V2 model assumes that the cytotoxic activity of CD8+ T cells within the tumor microenvironment does not change with time. In contrast, the V3 model assumes that, upon entering the tumor microenvironment, a subset of CD8+ T cells lose cytotoxic activity and an ability to produce IFNG, that is they become deactivated (Figure 5B). The ratio between the parameters *a*4 and *a*5 can be used to infer whether deactivation of CD8+ T cells is a constitutive process, as represented by parameter *a*4, or depends on a negative feedback mechanism mediated by IFNG or some other signal that is dependent on TILs, as represented by parameter *a*5. The parameter distributions suggest that deactivation of TILs primarily occurs through a negative feedback mechanism (log ratio *a*4/*a*5 = −3.2 ± 1.4), although very few CD8+ T cells that enter the tumor microenvironment become deactivated (P((*a*4 + *a*5)/(*kp*3*a* + *kd*5*a* + *a*4 + *a*5) > 0.5) < 0.1).

Given these changes in cytotoxic activity of CD8+ T cells, we also used the model to infer the fate of CD8+ T cells once they enter the tumor microenvironment, that is do they die, proliferate, or deactivate. The low degree of correlation among these parameters suggests that the parameters associated with these cell fate processes can be determined independently from the data (see Figures S3, S6). Specifically, we found that the rate constants associated with cell death (*kd*5) and proliferation (*kp*3*a*) of TILs independently increase when the antigen density increases but the balance between cell death and proliferation is different between the V2 and V3 models. Using pairwise scatter plots to highlight the differences (see Figures 5C,D), the ensemble of parameter values associated with the V2 and V3 models are clustered in the regions indicated by red and blue arrows, respectively. Results for both models show that the majority of values for the cell fate parameters are clustered below the diagonal suggesting that both *kd*5+ is greater than *kd*5− and *kp*3*a*+ is greater than *kp*3*a*−, where a minus (−) or a plus (+) appended to the parameter symbol indicates whether the parameter values were determined using the rHuAd5-hgp100 or rHuAd5-hDCT data, respectively. For the V2 model, *kp*3*a*− vs. *kd*5− is on diagonal while the cluster of parameter values associated with *kp*3*a*− vs. *kd*5− is above diagonal for the V3 model, which implies that *kp*3*a*− > *kd*5−. In an antigen-dense environment, *kp*3*a*+ vs. *kd*5+ is above the diagonal for the V2 model and while, for the V3 model, *kp*3*a*+ vs. *kd*5+ is below diagonal, which implies that *kd*5+ > *kp*3*a*+.

Collectively, results using the V2 model suggest that, in an antigen-sparse environment, CD8+ T cells that enter the tumor microenvironment are equally likely to die as to proliferate (log ratio *kp*3*a*− / *kd*5− = 0.2 ± 1.2). In an antigen-dense environment, CD8+ T cells are, on average, 40 times more likely to proliferate than die (log ratio *kp*3*a*+ / *kd*5+ = 1.6 ± 1.3). In contrast, interpreting the data using the V3 model suggests that CD8+ T cells that enter the tumor microenvironment are 12 times more likely to proliferate than die when antigen is sparse (log ratio *kp*3*a*− / *kd*5− = 1.1 ± 1.5) and, in an antigen-dense environment, are initially 4 times more likely to die than proliferate (log ratio *kp*3*a*− / *kd*5− = −0.63 ± 2.0). Once CD8+ T cells commit to a deactivated phenotype, they are twice as likely to proliferate than die (log ratio *kp*3*b* / *kd*5*b*+ = 0.29 ± 0.93). Collectively, interpreting the data using the V3 model suggests that both the rate coefficients for cell proliferation and cell death of deactivated *T*_*E*3_ (*T*_*E*3*b*_, *T*_*E*3*c*_, and *T*_*E*3*d*_) are greater than newly infiltrating TILs (*T*_*E*3*a*_) but the balance between cell fates is different such that the fate favors cell death of newly infiltrating TILs while deactivated states of *T*_*E*3_ favor cell proliferation.

### 3.4. Improving tumor control by CD8+ T cells was most sensitive to increasing MHC class I presentation of tumor antigens

To validate the V3 model, we used the model with the statistically sampled ensemble of parameter values to test whether the implied assumptions are consistent with experimental studies that use different experimental protocols. In particular, we simulated an adoptive cell therapy experiment reported by Zhou et al. where 3 × 10^6^ pmel-1 CD8 T cells were adoptively transferred into mice 10 and 15 days after intradermal challenge with 1 × 10^5^ B16 cells (Figure 6) (Zhou et al., 2014). As primary pmel-1 CD8+ T cells recognize the predominant immunogenic epitope derived from gp100 (Overwijk et al., 2003), modeling and simulation of rHuAd5-hgp100 immunotherapy in the B16 model was used as a basis for these adoptive cell transfer (ACT) experiments. Specifically, ACT was modeled by bypassing the lymph node compartment and introducing CD8+ T cells directly into the blood compartment, with the rest of the parameters remaining the same as calibrated. Given the average blood volume of a 10 week-old mouse, ACT of this number of CD8+ T cells corresponds to a blood concentration of 2500 cells per mm^3^ (Figure 6A). While CD4+ T cells were adoptively transferred simultaneously as the CD8+ T cells, the simulations incorporated just ACT of CD8+ T cells, as a slight inhibition of tumor growth was dependent on CD8+ T cells. To match the basal tumor growth rate, an initial bolus of 5 × 10^5^ B16 cells was used instead of the bolus of 1 × 10^6^ cells used in calibrating the model. Using model parameters for T cell dynamics associated with the rHuAd5-hgp100 experiments, the simulated ACT of gp100 CD8+ T cells provided a similar reduction in tumor size as experimentally observed (Figure 6B). These results validate that the inferred parameters for CD8+ T cell-mediated control of B16 tumors obtained by analyzing the rHuAd5-hgp100 data accurately simulate ACT of B16F10 tumors using primary pmel-1 CD8+ T cells.

To improve control of tumor growth, Zhou et al. identified a number of genes that, when knocked down, improve control of tumor growth. For instance, *Ppp2r2d* was identified in the in vivo screen that, when knocked down with shRNA, reduced tumor size (Figure 6B). As it is unclear as to the biological role that *Ppp2r2d* plays in regulating T cell function, Zhou et al. postulated that *Ppp2r2d* could play a number of roles in improving IFNG production by, reducing the propensity for apoptosis of, and increasing the proliferation rate of tumor infiltrating CD8+ T cells. As these three mechanisms are uniquely represented by different parameters in the mathematical model, we performed a sensitivity analysis to see how altering the corresponding parameters influences tumor size (Figure 6C). Two parameters, *kc*1 and *kp*3*a*, were increased by a factor of 10^5^ while the parameter corresponding to the rate of TIL cell death, *kd*5*a*, was reduced by a factor of 10^5^. Two thousand simulations were performed with an ensemble of parameter values obtained from the converged segments of the Markov Chains to generate a distribution in the tumor growth profiles in response to changing each of these three parameters in isolation and simultaneously. A snapshot of the tumor growth profiles obtained at day 19 were used for comparison. As summarized in Figure 6C, increasing IFNG production (*kc*1) slightly reduced tumor size (median for *kc*1: 86 mm^2^ vs. unmodified ACT of gp100 T cells: 92 mm^2^, *p*-value < 0.001) while the other two parameters had essentially no impact on tumor size (median for *kd*5*a*: 92 mm^2^ and *kp*3*a*: 91 mm^2^, *p*-values vs. unmodified both > 0.05). In addition, altering all three parameters simultaneously had no added benefit as the slight reduction in tumor size was the same as increasing *kc*1 in isolation (median for all three: 86 mm^2^).

As none of the mechanisms proposed by Zhou et al. seemed to mimic the observed reduction in tumor size, we explored alternative hypotheses as to how tumor size could be reduced following ACT of gp100 CD8+ T cells. In particular, we tested three alternative hypotheses. First, we increased the retention of TILs within the tumor microenvironment by altering the preference of gp100 CD8+ T cells for the tumor microenvironment, that is we decreased *K*32 by a factor of 10^5^. Increasing the retention of TILs reduced tumor size slightly (median for *K*32: 80 mm^2^). Second, we decreased the number of deactivated TILs in two ways by setting the parameters *c*4*b*, *c*4*c*, and *c*4*d* equal to *c*4*a* and by reducing the parameters *a*4 and *a*5 by a factor of 10^5^. Decreasing the number of deactivated TILs had essentially no impact on tumor size (median for *c*4's equal: 89 mm^2^ vs. *a*4 and *a*5: 91 mm^2^). The third alternative hypothesis was to increase the parameter associated with converting antigen-deficient B16 tumor cells to B16 cells that present antigen recognized by TILs, that is *c*3. Increasing MHC class I presentation had a more pronounced effect on tumor growth such that an increase by a factor of 10 was sufficient to eliminate B16 tumor cells (median for *c*3 decreased by a factor of 3: 12 mm^2^ and by a factor of 10: 0.02 mm^2^). In both cases, increasing antigen presentation was predicted to reduce tumor size out to 50 days, where the median tumor size for increasing *c*3 by a factor of 3 was 6 mm^2^ (1st quartile: 1 mm^2^ and 3rd quartile: 13,000 mm^2^) and by a factor of 10 was 0 mm^2^ (1st and 3rd quartiles: 0 mm^2^).

As the B16 cell line is known to have defects in the processing and presentation of gp100 (Leitch et al., 2004), we also simulated the adoptive cell transfer of CD8+ T cells that recognize epitopes derived from DCT. While ACT using DCT CD8+ T cells reduced tumor size relative to improving the retention of TILs (ACT of DCT T cells: 71 mm^2^), improving antigen presentation by B16 provided the best control of tumor growth. As the *in vivo* experiments include additional cellular components than included in the mathematical models, the results suggest that altering *Ppp2r2d* may influence the control of tumor growth through an indirect mechanism. One possibility is that an increase in IFNG production may increase antigen presentation by tumor cells indirectly by inhibiting the action of myeloid-derived suppressor cells.

### 3.5. *In vivo*, B16 tumors include time-dependent factors that limit the efficacy of anti-tumor immunity

As a common criticism of testing immunotherapies using syngeneic animal models is that the timing of the initiation of immunotherapy influences the control of tumor growth (Wen et al., 2012). For instance, an early pre-clinical study using a monoclonal antibody against cytotoxic T lymphocyte antigen (CTLA)-4 in conjunction with a granulocyte/macrophage colony-stimulating factor (GM-CSF) producing irradiated tumor cell vaccine found that initiation of the therapy within 4 days of intradermal challenge with a variant of the B16 cell line was able to control tumor growth while initiation of the therapy after 8 days was less effective and essentially ineffective after 12 days (van Elsas et al., 1999). The observed therapeutic effect requires generating CD8+ T cells against tumor antigens. A number of possible explanations for the time-dependent loss of efficacy was proposed. For instance, the magnitude of the immune response initiated by the therapy may be unable to control tumor growth at the later time points as tumors grow above a critical size. Alternatively, the loss of efficacy may be a consequence of tumor-induced deletion of T cells that recognize tumor-associated antigens. Finally, the tumor microenvironment may change as a function of time such that initially the microenvironment permits but, in time, develops an immunosuppressive environment that suppresses a cell-mediated anti-tumor immune response. *In vivo* these different mechanisms are intimately linked and are difficult to test in isolation. Given that complex dynamic systems respond in non-linear ways to perturbations, modeling and simulation can provide insight into the contributions of these different mechanisms.

Next, we used the V3 model to test whether changing the timing of rHuAd5-hDCT therapy changes the dynamics of tumor growth similarly to that observed by Allison and coworkers (van Elsas et al., 1999) (Figure 7). First, we used the parameter values for the V3 model to simulate the experiment but reduced the bolus of intradermal B16 challenge by half to match the early tumor growth trajectory observed in mice treated with an IgG control (Figure 7A). Interestingly, the observed growth of B16 tumors in mice treated with IgG appears to level off at a tumor size of 350 mm^2^, suggesting that an increase in tumor size limits the proliferation rate of tumor cells and the maximum tumor size is referred to as the carrying capacity. In calibrating and validating the model, incorporating a carrying capacity was not necessary as the maximum tumor sizes where lower at approximately 250 mm^2^. As the model was not designed to capture tumor growth around the carrying capacity, we focused on comparing the model predictions and data below the carrying capacity. Next, we changed the timing of rHuAd5-hDCT therapy such that the immunotherapy was initiated 0, 5, and 10 days following intradermal challenge of B16F10 cells (Figures 7B,C). As the immunization was delayed, the peak in the number of CD8+ T cells in the blood shifted to longer times but, interestingly, the maximum number of CD8+ T cells in the blood decreased (Figure 7B). As the number of CD8+ T cells in the blood is a dynamic equilibrium with the tumor compartment, more CD8+ T cells migrate to the tumor compartment as the compartment size increases thereby decreasing the number of CD8+ T cells in the blood. In terms of tumor growth, the simulated profiles exhibited similar dynamics. Initiation of rHuAd5-hDCT earlier was able to sustain a reduced rate of tumor growth for longer but, as the number of CD8+ T cells in the system declined, tumor growth returned to the same exponential growth rate. Even if the number of TILs is increased, either through reducing the negative feedback on clonal expansion of CD8+ T cells in the lymph node (e.g., by decreasing *ka*) or adoptively transferring 100 times more DCT CD8+ T cells (see Figure 6), a further reduction in tumor growth is limited as the conversion of MHC class I negative to MHC class I positive cells (e.g., *c*3) becomes the rate limiting step for controlling tumor growth (see Figure 6C). Increasing the rate constant associated with MHC class I conversion by a factor of 3 (e.g., 3 × *c*3) in conjunction with immunizing at day 10 was still effective (median tumor size at day 50 with immunizing at day 0: 0.1 mm^2^, vs. at day 10: 1.3 mm^2^). The day 0 plus 3 × *c*3 results are shown in Figure 7C, while results for day 10 plus 3 × *c*3 are not shown as they essentially overlap. In summary, changing the timing for immunization was unable to control of tumor growth. Capturing total control of tumor growth, as observed for the Day 0 and Day 4 conditions, was dependent on modifying the model in two ways: by increasing the number of tumor-infiltrating CD8+ T cells that recognize tumor associated antigens and by increasing antigen presentation by tumor cells. In comparing the observed (Figure 7A) vs. simulated (Figure 7C) tumor growth profiles, the observed tumor growth curves have different trajectories. Total control of tumor growth characterizes the early time points (Day 0 and 4). In time, total control transitions to partial control, which is characterized by a progressive increase in tumor growth rate but also an increase in steady-state tumor burden. Collectively, the results are consistent with a model where immunotherapy can generate a sufficient CD8+ T cell response throughout the 12-day timeframe to control tumor growth that initially coincides with an increase in antigen presentation by tumor cells. However, this increase in antigen presentation by tumor cells is lost between days 4 and 12. Moreover, the loss of antigen presentation is independent of changes in IFNG within the tumor microenvironment as the sensitivity analysis results suggest that the effect of IFNG is saturated in immunized animals.

## 4. Discussion

Identifying local barriers that limit the efficacy of anti-tumor immunity is a critical barrier for broadening the clinical benefit of immunotherapies for cancer. Given the challenges associated with testing hypotheses in humans, pre-clinical mouse models, like the B16 model for malignant melanoma, play a central role in identifying how tumors escape from immune-mediated control. To gain insight into potential key mechanisms associated with the control of B16F10 tumor growth by CD8+ T cells, we combined dynamic observations of CD8+ T cell response and tumor growth following an adenovirus-based vaccination against tumor antigens with mechanistic multi-scale modeling and simulation.

The key conceptual shift here is the focus on whether the postulated network topology of the model can describe the observed data by eliminating the confounding uncertainty associated with the model parameters. This was done by using a Markov Chain Monte Carlo approach to obtain a large statistical sample of the parameter values that provide predictions similar to the observed data. To illustrate the approach, we asked two questions. First, we asked whether the postulated network topology, that is the modeled biological components and their associated interactions, is consistent with the observed data. Drawing from a base model (V1), a new model (V2) was proposed to better capture observed differences between three different experimental conditions. To weight the evidence in support of the competing models, Bayes Ratios suggested that the data provide strong to very strong evidence in favor of the V2 over the V1 model. In addition, we assumed that the phenotype of tumor infiltrating lymphocytes is conserved, but models incorporating this assumption (V1 and V2) were unable to capture observed changes in IFNG gene expression. In a revised model (V3), we assumed that the phenotype of tumor infiltrating lymphocytes progressively changes, as modeled by a decrease in cytotoxic killer function and IFNG production. Here, Bayes Ratios suggested that the data provide weak but supportive evidence in favor of the modifications included in the V3 relative to the V2 models. In addition, trying to capture the IFNG response suggested specific experimental conditions where additional experiments could help refine our understanding of the dynamic response of CD8+ T cells to tumor localization.

The second question was whether we could infer the relative importance of the different mechanisms associated with the regulation and activity of tumor infiltrating lymphocytes, given the available data. Using differences in the posterior distributions of model parameters, the fate of CD8+ T cells upon entering the tumor microenvironment changed depending on the antigen density and activation status. For instance, newly infiltrating TILs prefer to die in an antigen dense environment while those that survive and become deactivated prefer to proliferate. As a result of stronger TCR engagement in the absence of adequate costimulation, the fate of newly infiltrating TILs is consistent with activation-induced cell death followed by inducing T cell exhaustion in surviving cells (Wherry and Kurachi, 2015). In contrast, newly infiltrating TILs prefer to proliferate in an antigen-sparse environment. In both cases, TILs deactivate preferentially through a negative feedback mechanism related to ongoing anti-tumor immunity. The tumor antigens selected as immunogen had an impact on control of tumor growth as immunizing against hDCT expanded CD8+ T cells with a cytotoxic killing efficacy of 10,000 times higher than CD8+ T cells that recognize epitopes derived from hgp100. However, the impact of both therapies were limited such that effective elimination of existing B16F10 tumors also depended on an increase in MHC class I presentation of tumor antigens. *In vivo*, observed changes in efficacy of an immunotherapy administered at different days following subcutaneous tumor challenge was consistent with a progressive loss in time of MHC class I antigen presentation, which was largely independent of changes in IFNG within the tumor microenvironment and the number of TILs. In short, increasing the number of TILs that recognize tumor antigens was necessary but not sufficient to control the growth of B16F10 tumors effectively.

Loss of MHC class I antigen presentation is a common mechanism whereby both solid and hematological tumors escape immune-mediated control of malignant cell outgrowth (Campoli and Ferrone, 2008). This loss of MHC class I antigen presentation can be attributed to either genetic alterations, which remain conserved within the time window used for transplantable mouse models, or transient epigenetic regulation. Here, the simulation results suggest that, between day 4 and day 8, B16 tumors lose their ability to present tumor antigens via MHC class I proteins, which results in escape from CD8+ T cell-mediated control of tumor growth. In this context, IFNG is thought to be critical in regulating MHC class I protein expression by tumor cells. *In vitro*, an increase in IFNG induces many melanoma cell lines to upregulate MHC class I expression (Mendez et al., 2008). *In vivo*, antibody blockade and knock-out of IFNG abrogates any benefit of immunization in controlling B16F10 tumor growth (McGray et al., 2014). However, the simulation results suggest that the effect of IFNG release within the tumor microenvironment is saturated, therefore further increases in IFNG will not reduce tumor growth. Collectively, the results suggest a transient disconnect between the extracellular signal, that is TILs release of IFNG, and the tumor response.

The transient disconnect between IFNG signal and tumor response could occur in a number of different ways. One mechanism is through epigenetic regulation of the tumor response via *de novo* methylation of DNA linked with MHC class I expression (Khan et al., 2008). For instance, RNAi mediated knock-down of Dnmt3a, a DNA methyltransferase, had no effect on the in vitro phenotype of B16 cells but increased the expression of genes associated with MHC class I antigen processing and presentation and dramatically reduced tumor growth *in vivo* (Deng et al., 2009). Expression of Dnmt3a has been linked to epithelial-mesenchymal transition (EMT), where miRNAs that regulate Dnmt3a expression are lost upon EMT and enable de novo methylation to increase (Cicchini et al., 2015). In turn, EMT is induced by hypoxia (Cooke et al., 2012), which can become more prevalent within the tumor microenvironment as tumors increase in size. Alternatively, the cellular composition within the tumor microenvironment changes following subcutaneous challenge, which can alter peptide presentation. For instance, myeloid-derived suppressor cells accumulate within the tumor microenvironment and produce the free radical peroxynitrite that can nitrate MHC class I molecules, which inhibits the binding and retention of processed peptides to tumor cell-associated MHC proteins (Lu et al., 2011).

Collectively, this work represents one iteration of a design-build-test cycle, where experimental observations provide design constraints for the model, a network topology is proposed to capture the observed behaviors, and the testing stage involves evaluating the fitness of the math model to capture the observed data. A common criticism of mathematical models is that biological components, like myeloid-derived suppressor cells (Marvel and Gabrilovich, 2015; Parker et al., 2015), or interactions between these components, like suppression of cell proliferation by IFNG produced by CD8+ T cells (Matsushita et al., 2015), thought to play important roles within the tumor microenvironment are not explicitly represented by the model topology. While *in vivo* models naturally incorporate these network elements, the relative contributions of these elements *in vivo* can subtly change in non-intuitive ways depending on context, which can depend on the experimental design. When these elements change simultaneously, it is difficult to parse the unique contributions of each of these elements in coordinating system response. Ultimately, the goal is to understand how these elements causally influence the system's response and how these elements can be manipulated for therapeutic aims. As part of an iterative approach where the complexity of the model is progressively increased or competing hypotheses are tested, mathematical modeling and simulation plus targeted experiments provide a more powerful approach to think more clearly about how a biological network becomes altered in disease and how this altered network can be restored using therapeutic means.

## Author contributions

DK conceived the study, DK, QW developed the mathematical model, DK, QW performed the data analysis, DK drafted the manuscript, DK, QW revised the manuscript, and DK, QW approved the final version.

## Funding

This work was supported by grants from the National Science Foundation (CAREER 1053490 to DK), the National Cancer Institute (NCI) (R15CA123123 and R01CA193473 to DK), and the National Institute of General Medical Sciences (NIGMS) as part of the WV-INBRE (P20GM103434 to QW). The content is solely the responsibility of the authors and does not necessarily represent the official views of the NCI, NIGMS, the National Institutes of Health, or the National Science Foundation. The funders had no role in study design, data collection and analysis, decision to publish, or preparation of the manuscript.

### Conflict of interest statement

The authors declare that the research was conducted in the absence of any commercial or financial relationships that could be construed as a potential conflict of interest.
