# Supplementary material for: Inferring the Impact of Regulatory Mechanisms that Underpin CD8+ T Cell Control of B16 Tumor Growth In vivo Using Mechanistic Models and Simulation
Source: Front Pharmacol. 2017 Jan 4;7:515. doi: 10.3389/fphar.2016.00515 (PMC5209634; doi:10.3389/fphar.2016.00515)
Supplement: Supplementary file 1 [file DataSheet1.PDF]

Supplementary Material: Inferring the impact of regulatory mechanisms that underpin CD8+ T cell control of B16 tumor growth in vivo using mechanistic models and simulation

David J. Klinke II* and Qing Wang

*Correspondence:
David J. Klinke II:
david.klinke@mail.wvu.edu

1 SUPPLEMENTARY TABLES AND FIGURES

This PDF file includes:

Table S1 List of parameters for V2 and V3 models and their corresponding maximum expectation values.
Table S2 List of the V2 and V3 model parameters that change for different experimental conditions and corresponding maximum expectation values.
Fig. S1. Convergence of AMCMC results for the V2 model.
Fig. S2. AMCMC summary plots for each of the model parameters associated with the V2 model.
Fig. S3. Pairwise comparison of posterior distribution in parameter values corresponding to the V2 model.
Fig. S4. Convergence of AMCMC results for the V3 model.
Fig. S5. AMCMC summary plots for each of the model parameters associated with the V3 model.
Fig. S6. Pairwise comparison of posterior distribution in parameter values corresponding to the V3 model.
References

Table S1. List of parameters for V2 and V3 models and their corresponding maximum expectation values.

Symbol	Definition	V2 Model Value	V3 Model Value	Units
C_{MHCI-}	Initial value of MHC class I negative tumor volume	1.52	1.28	mm^3
T_{E2T}	Total number of CD8+ T cells in the blood (2)	5.69×10^5	5.69×10^5	$cells \cdot mm^{-3}$
k_{d1}	Naïve CD8+ T cell natural death rate constant	1.77×10^{-2}	1.02×10^{-1}	day^{-1}
k_{d2}	Adenovirus decay rate constant	0.65	0.65	day^{-1}
k_{d3}	T_{E2} decay rate constant	9.35×10^{-2}	9.68×10^{-2}	day^{-1}
k_{d4}	Rate constant for basal tumor cell death	6.46×10^{-2}	4.53×10^{-5}	day^{-1}
k_{d6}	IFNG decay rate constant	1.79×10^{-7}	9.99×10^{-8}	day^{-1}
k_{d7}	TNFα decay rate constant	2.30×10^{-6}	2.73×10^{-7}	day^{-1}
k_{p1}	Rate constant for effector CD8+ T cell proliferation in lymph node due to adenovirus vaccination	1.44	1.53	day^{-1}
k_{p2}	Rate constant for tumor cell proliferation	5.02×10^{-1}	4.46×10^{-1}	day^{-1}
a_{12}	Rate constant for CD8+ T cell transport from lymph node to blood	0.24	0.28	day^{-1}
K_{21}	Equilibrium distribution of CD8+ T cell in lymph node relative to blood (a_{21}/a_{12})	6.15×10^{-1}	8.20×10^{-1}	$unitless$
a_{23}	Rate constant for CD8+ T cell transport from blood to tumor	2.06×10^5	4.07×10^5	day^{-1}
a_4	Rate constant for constitutive deactivation of T_{E3}	–	1.49×10^{-7}	day^{-1}
a_5	Rate constant for deactivation of T_{E3} by IFNG	–	9.01×10^{-6}	day^{-1}
c_1	Synthesis rate for naïve CD8+ T cells by thymus	2.72×10^{-4}	2.72×10^{-4}	$cells \cdot mm^{-3} \cdot day^{-1}$
c_2	Rate constant for naïve CD8+ T cell activation in lymph node	9.34	7.75×10^4	day^{-1}
c_3	Rate constant for IFNG-dependent conversion of tumor cells from MHC class I negative to positive	1.76×10^{-1}	1.79×10^{-1}	day^{-1}
k_1	EC50 of IFNG	1.36×10^1	1.34×10^1	$moles \cdot mm^{-3}$
k_2	EC50 for autocrine-induced TNFα production	3.07	4.75	$moles \cdot mm^{-3}$
k_{c1}	Rate constant for IFNG production by effector CD8+ T cells in tumor microenvironment	1.13×10^1	6.23	$moles \cdot cell^{-1} \cdot day^{-1}$
k_{c2}	Rate constant for autocrine-inducible production of TNFα by effector CD8+ T cells in tumor microenvironment	5.98×10^3	1.17×10^5	$moles \cdot cell^{-1} \cdot day^{-1}$
k_{c3}	Rate constant for basal production of TNFα by effector CD8+ T cells in tumor microenvironment	2.21×10^{-3}	7.79×10^{-1}	$moles \cdot cell^{-1} \cdot day^{-1}$
k_g	Effective concentration of adenovirus antigen expression whereby 50% of CD8+ T cells that recognize immunogen are activated	4.34×10^3	4.69×10^3	$RLU \cdot mm^{-3}$
k_a	Effective concentration of effector CD8+ T cells in lymph node (T_{E1d}) whereby antigen-dependent proliferation of CD8+ T cells is inhibited by 50%	9.30×10^{10}	1.40×10^{11}	$cells \cdot mm^{-3}$

Table S1 - continued.

Symbol	Definition	V2 Model Value	V3 Model Value	Units
V_{ln}	Volume of lymph node from (3)	0.25	0.25	mm^3
V_b	Volume of blood	1.40×10^3	1.40×10^3	mm^3
V_i	Volume of immune cell from (1)	1.00×10^{-7}	1.00×10^{-7}	$mm^3 \cdot cell^{-1}$
β_1	Scaling constant for RT-PCR assay of $TCR\alpha$ expression	1.86×10^{-2}	1.71×10^{-2}	AU[$]
β_2	Scaling constant for RT-PCR assay of $IFN\gamma$ expression	8.55×10^{-4}	1.77×10^{-3}	AU
β_3	Scaling constant for RT-PCR assay of $TNF\alpha$ expression	2.74×10^{-6}	1.79×10^{-7}	AU
BG_1	Non-specific background for RT-PCR assay of $TCR\alpha$ expression	8.80×10^{-2}	5.90×10^{-2}	AU
BG_2	Non-specific background for RT-PCR assay of $IFN\gamma$ expression	2.55×10^{-3}	2.63×10^{-3}	AU
BG_3	Non-specific background for RT-PCR assay of $TNF\alpha$ expression	6.90×10^{-2}	6.73×10^{-2}	AU

[$] AU = arbitrary units

Table S2. List of the V2 and V3 model parameters that change for different experimental conditions and corresponding maximum expectation values.

Symbol	Definition	V2 Model Value	V3 Model Value	Units
	**Unstimulated control C57Bl/6 mice with B16F10 tumors**			
c_{4a}	Baseline cytotoxic activity of T_{E3a}	3.71×10^{-4}	1.80×10^{-5}	$mm^3 \cdot cells^{-1} \cdot day^{-1}$
c_{4b}	Baseline cytotoxic activity of T_{E3b}	–	2.57×10^{-9}	$mm^3 \cdot cells^{-1} \cdot day^{-1}$
c_{4c}	Baseline cytotoxic activity of T_{E3c}	–	2.79×10^{-13}	$mm^3 \cdot cells^{-1} \cdot day^{-1}$
c_{4d}	Baseline cytotoxic activity of T_{E3d}	–	3.97×10^{-17}	$mm^3 \cdot cells^{-1} \cdot day^{-1}$
k_{d5a}	T_{E3a} decay rate constant	2.78×10^{-5}	6.53×10^{-8}	day^{-1}
k_{d5b}	Rate constant for decay of T_{E3b}, T_{E3c}, and T_{E3d}	–	6.53×10^{-8}	day^{-1}
k_{p3a}	Rate constant for T_{E3a} proliferation	2.38×10^{-5}	7.98×10^{-7}	day^{-1}
k_{p3b}	Rate constant for T_{E3b}, T_{E3c}, and T_{E3d} proliferation	–	7.98×10^{-7}	day^{-1}
K_{32}	Equilibrium distribution of CD8+ T cells in blood relative to tumor (a_{32}/a_{23})	4.20	5.34	$unitless$
	**C57Bl/6 mice with B16F10 tumors immunized with rHuAd5-hgp100**			
c_{4a}	Cytotoxic activity of T_{E3a} against gp100$^+$ tumor cells	3.71×10^{-4}	1.80×10^{-5}	$mm^3 \cdot cells^{-1} \cdot day^{-1}$
c_{4b}	Cytotoxic activity of T_{E3b} against gp100$^+$ tumor cells	–	2.57×10^{-9}	$mm^3 \cdot cells^{-1} \cdot day^{-1}$
c_{4c}	Cytotoxic activity of T_{E3c} against gp100$^+$ tumor cells	–	2.79×10^{-13}	$mm^3 \cdot cells^{-1} \cdot day^{-1}$
c_{4d}	Cytotoxic activity of T_{E3d} against gp100$^+$ tumor cells	–	3.97×10^{-17}	$mm^3 \cdot cells^{-1} \cdot day^{-1}$
k_{d5a}	T_{E3a} decay rate constant	2.78×10^{-5}	6.53×10^{-8}	day^{-1}
k_{d5b}	Rate constant for decay of T_{E3b}, T_{E3c}, and T_{E3d}	–	6.53×10^{-8}	day^{-1}
k_{p3a}	Rate constant for T_{E3a} proliferation	2.38×10^{-5}	7.98×10^{-7}	day^{-1}
k_{p3b}	Rate constant for T_{E3b}, T_{E3c}, and T_{E3d} proliferation	–	7.98×10^{-7}	day^{-1}
K_{32}	Equilibrium distribution of CD8+ T cells in blood relative to tumor (a_{32}/a_{23})	4.20	5.34	$unitless$
	**C57Bl/6 mice with B16F10 tumors immunized with rHuAd5-hDCT**			
c_{4a}	Cytotoxic activity of T_{E3a} against DCT$^+$ tumor cells	3.18×10^{1}	8.05×10^{1}	$mm^3 \cdot cells^{-1} \cdot day^{-1}$
c_{4b}	Cytotoxic activity of T_{E3b} against DCT$^+$ tumor cells	–	1.14×10^{-2}	$mm^3 \cdot cells^{-1} \cdot day^{-1}$
c_{4c}	Cytotoxic activity of T_{E3c} against DCT$^+$ tumor cells	–	1.25×10^{-6}	$mm^3 \cdot cells^{-1} \cdot day^{-1}$
c_{4d}	Cytotoxic activity of T_{E3d} against DCT$^+$ tumor cells	–	2.49×10^{-10}	$mm^3 \cdot cells^{-1} \cdot day^{-1}$
k_{d5a}	T_{E3a} decay rate constant	1.42×10^{-3}	2.40×10^{-2}	day^{-1}
k_{d5b}	Rate constant for decay of T_{E3b}, T_{E3c}, and T_{E3d}	–	8.98×10^{1}	day^{-1}
k_{p3a}	Rate constant for T_{E3a} proliferation	2.83×10^{-1}	2.94×10^{-6}	day^{-1}
k_{p3b}	Rate constant for T_{E3b}, T_{E3c}, and T_{E3d} proliferation	–	5.78×10^{-2}	day^{-1}
K_{32}	Equilibrium distribution of CD8+ T cells in blood relative to tumor (a_{32}/a_{23})	1.69	1.64	$unitless$

1.1 Figures

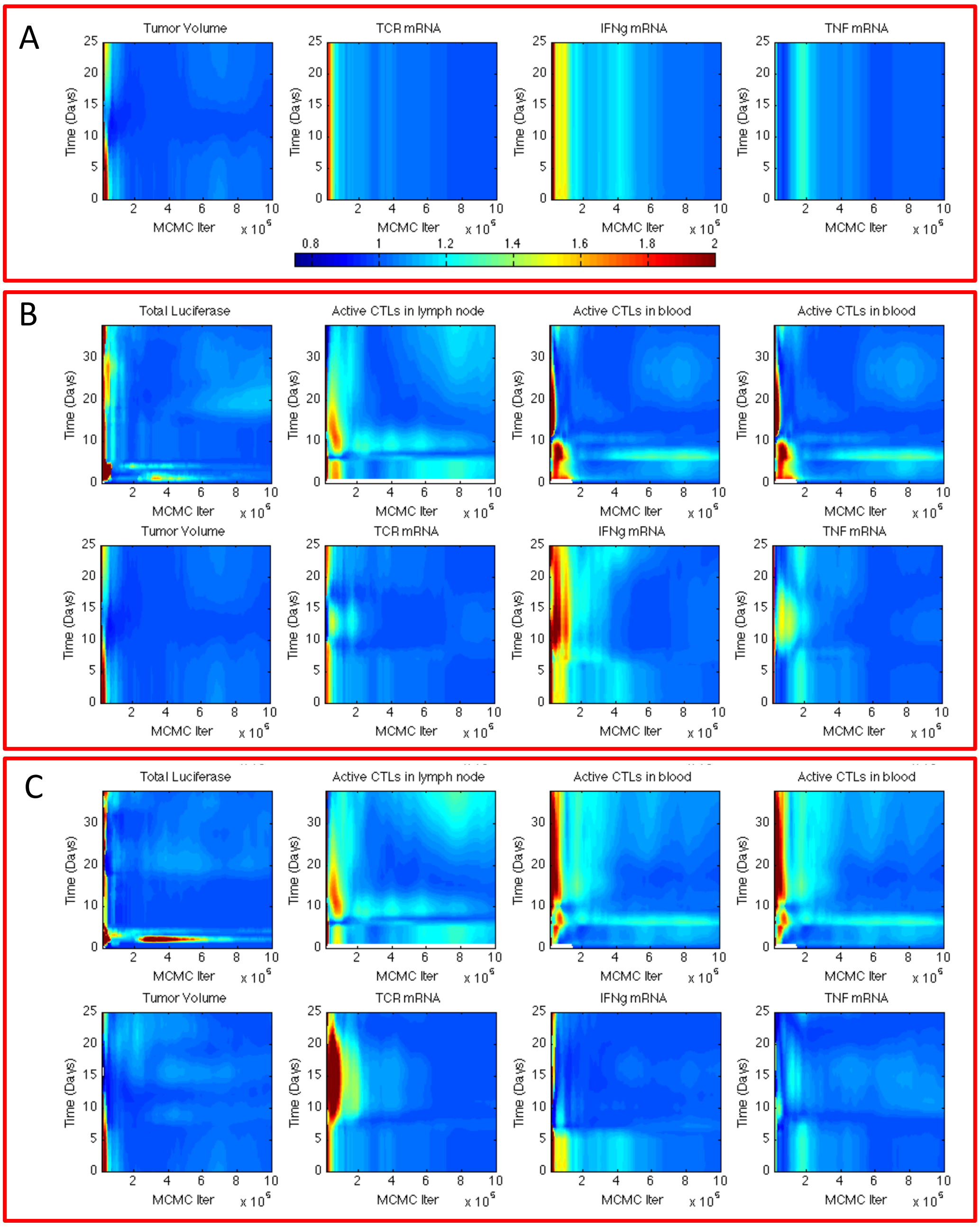

Figure S1. Convergence of AMCMC results for the V2 model. A contour plot of the Gelman-Rubin potential scale reduction factor (PSRF) of the model predictions shown as functions of time (y-axis) and AMCMC step (x-axis). Four parallel chains were used to calculate the Gelman-Rubin statistics for the model-based inference of the observed biological response following implantation of B16F10 tumors in C57Bl/6 mice that were untreated (A) or immunized with either rHuAd5-hgp100 (B) or rHuAd5-hDCT (C). For untreated mice, the simulated responses include tumor volume and mRNA for TCR, IFNG, and TNFα. For immunized mice, the simulated responses include total luciferase, active CD8+ T cells (CTLs) in the lymph node, active CTLs in blood, tumor volume, and mRNA for TCR, IFNG, and TNFα. PSRF values less than 1.2 suggest convergence of the chains.

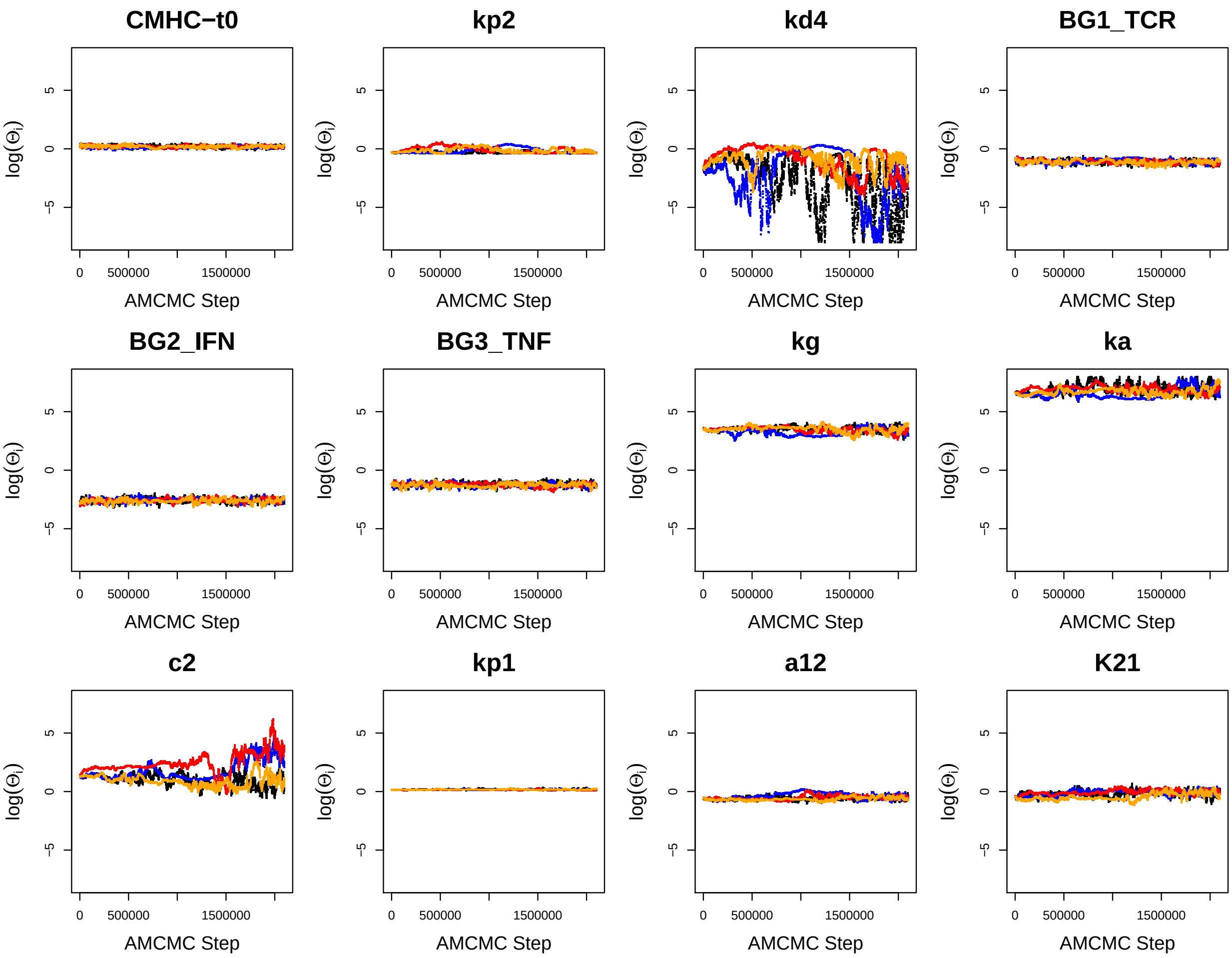

Figure S2. AMCMC summary plots for each of the model parameters associated with the V2 model. The trace of each of the model parameters is shown as a function of AMCMC step, where the parameter name is indicated above the panel. The traces for four parallel chains are shown in different colors: chain 1 (blue), chain 2 (black), chain 3 (red), and chain 4 (orange). The last two panels are the trace of the posterior likelihood ($P(Y|M, \theta)$) for each of the AMCMC chains with respect to data from untreated and rHuAd5-hgp100 conditions.

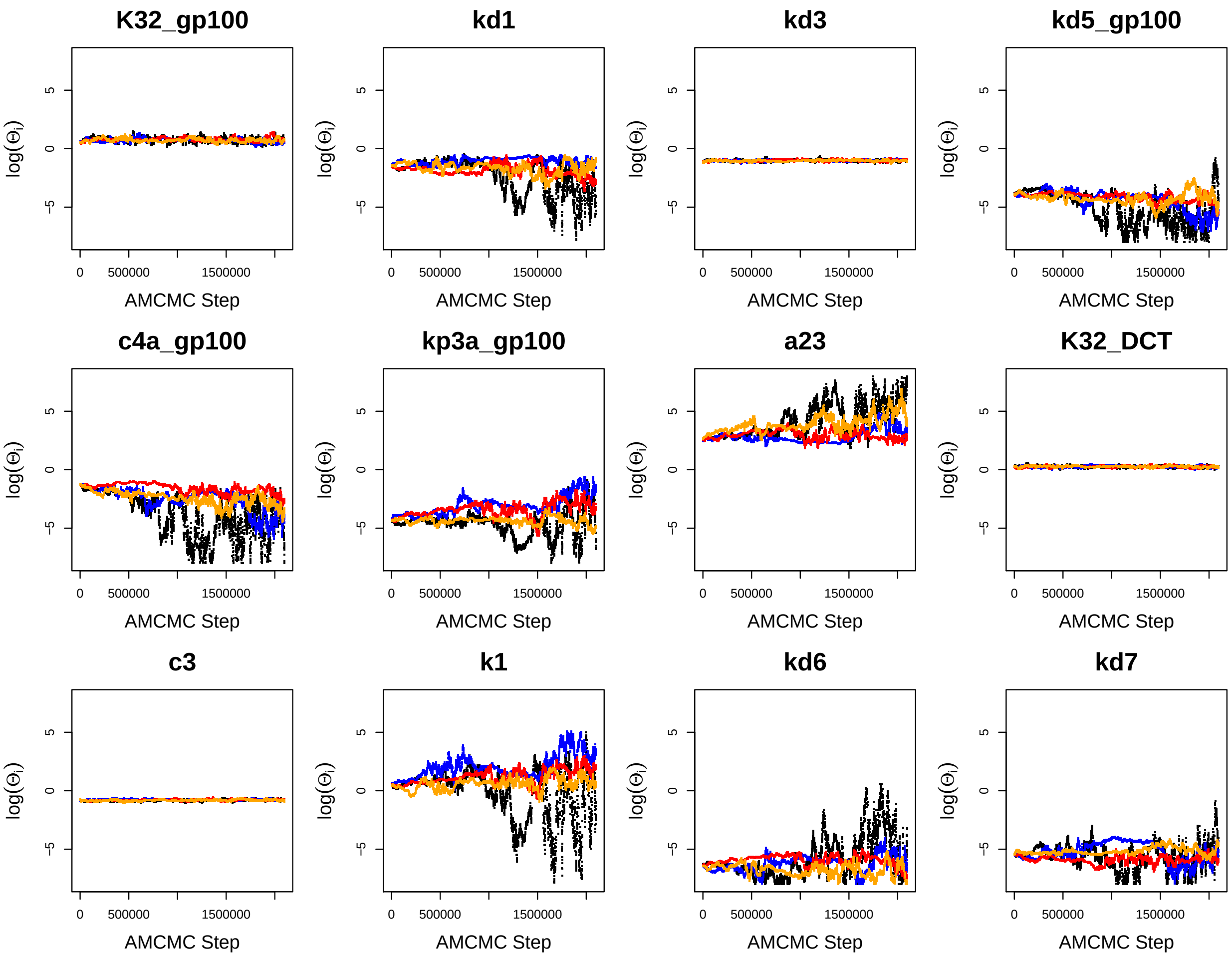

Figure S2 - continued.

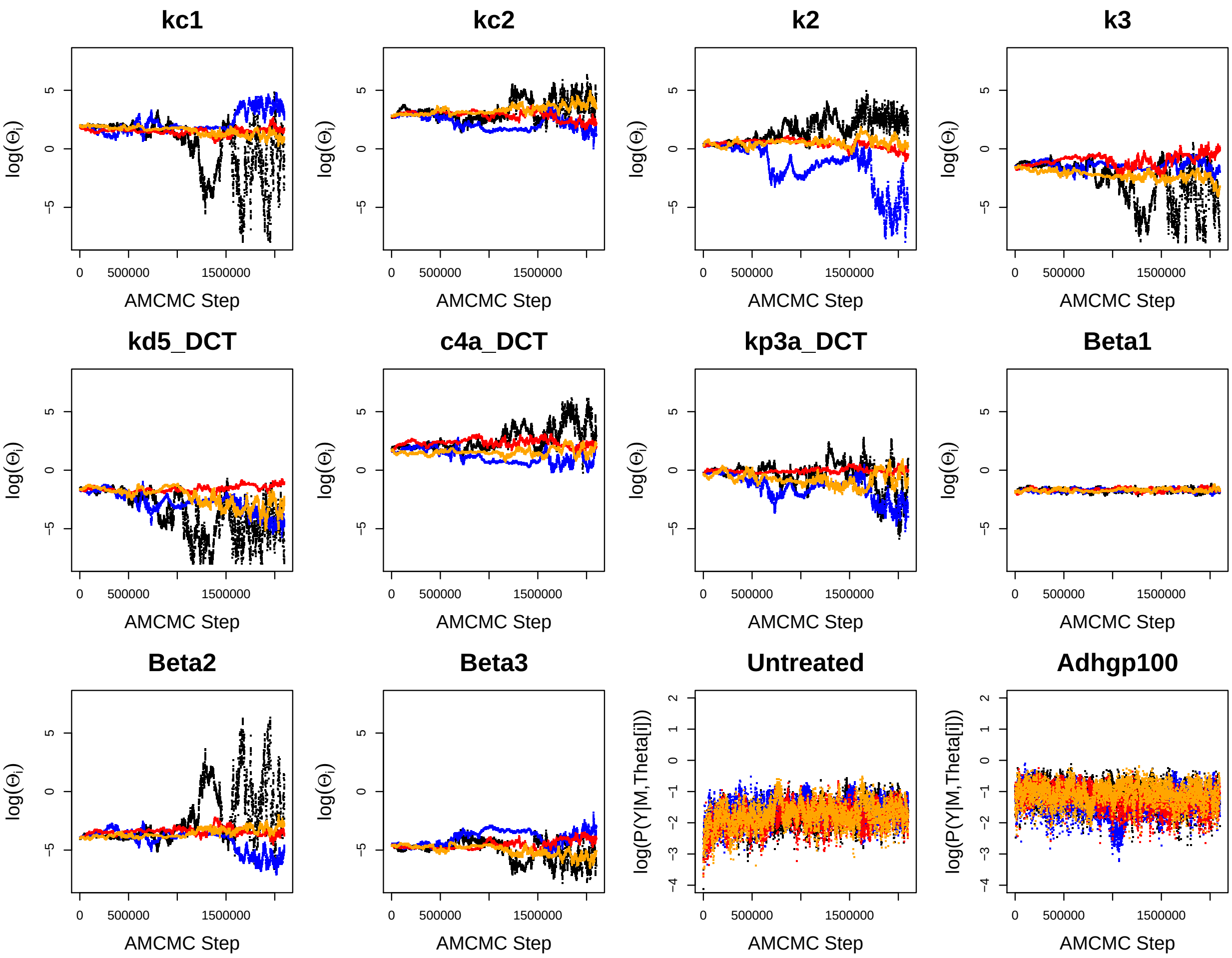

Figure S2 - continued.

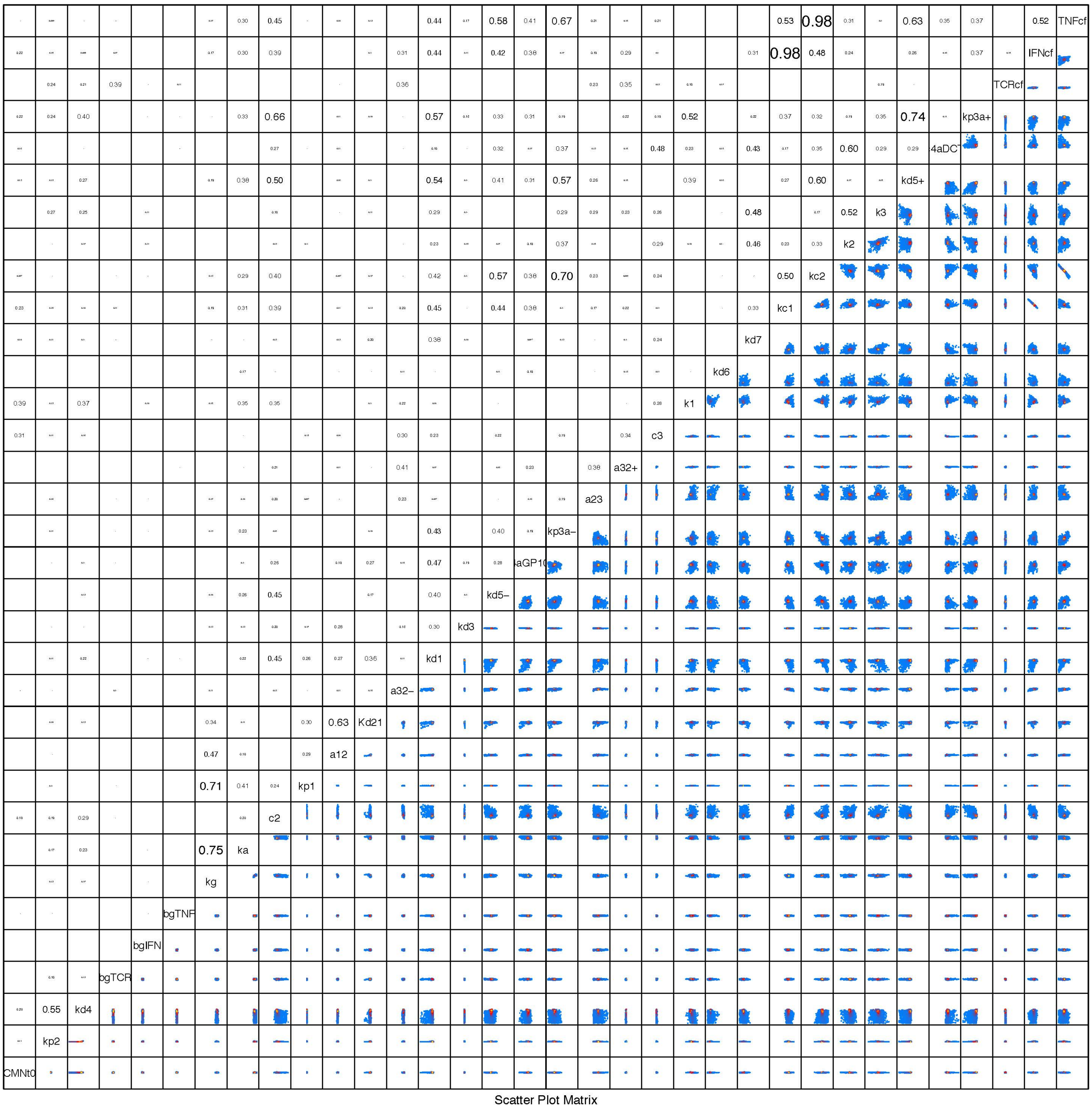

Figure S3. Pairwise comparison of posterior distribution in parameter values corresponding to the V2 model. Parameter names are given on the diagonal. Above the diagonal are the pairwise correlation coefficients of the parameters obtained from the four thinned Markov chains, where the font size is proportional to the value of the correlation coefficient. Pairwise projections of the marginalized probability density in $\log_{10}$ space are given below the diagonal. Coloring is based upon the estimated 2-D posterior density distributions using kernel density estimation. The axes for the scatter plots each spans from 10^{-8} to 10^{8}.

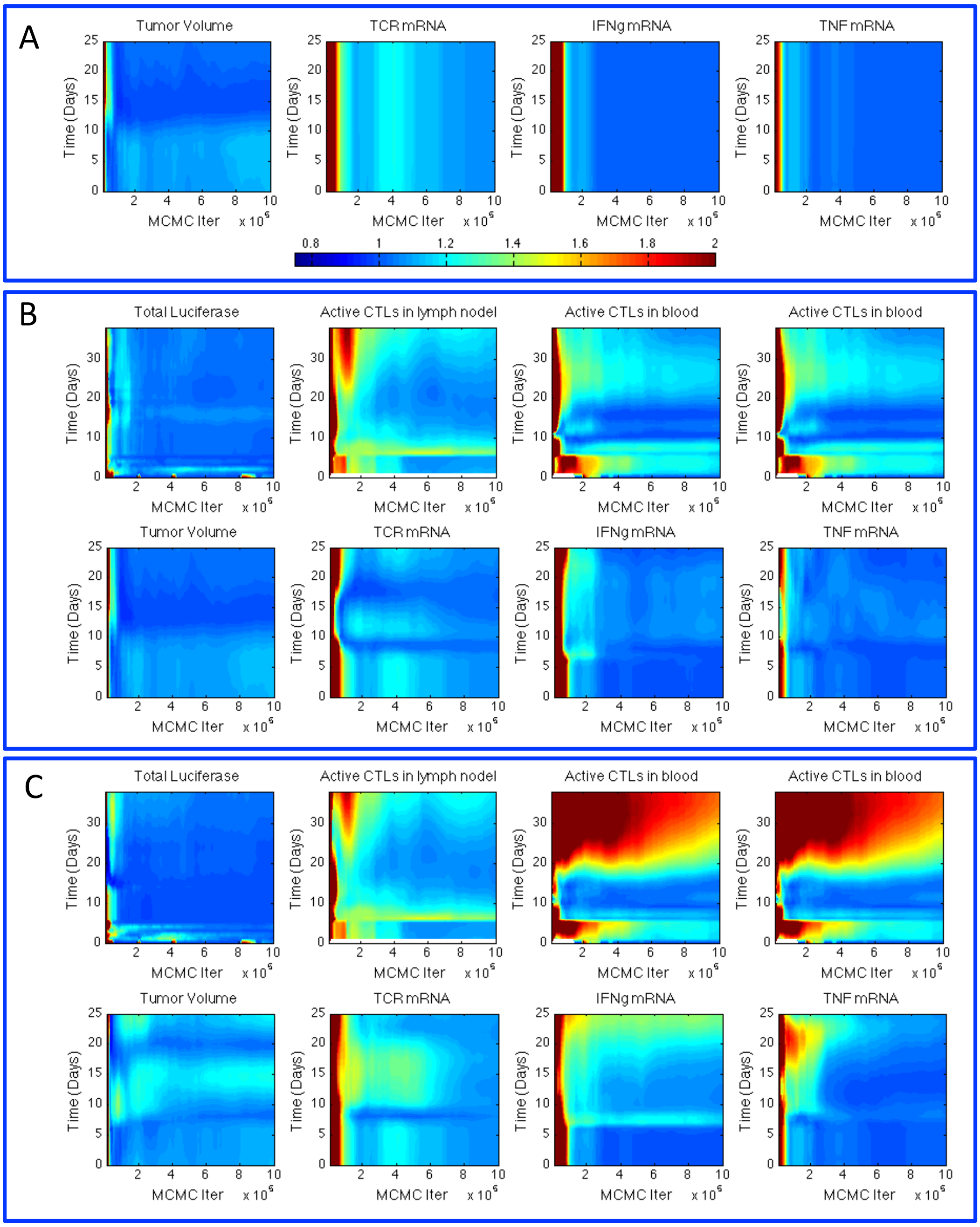

Figure S4. Convergence of AMCMC results for the V3 model. A contour plot of the Gelman-Rubin potential scale reduction factor (PSRF) statistic of the model predictions shown as functions of time (i.e., the y-axis) and AMCMC step (i.e., the x-axis). Four parallel chains were used to calculate the Gelman-Rubin statistics for the model-based inference of the observed biological response following implantation of B16F10 tumors in C57Bl/6 mice that were untreated (A) or immunized with either Adhgp100 (B) or AdhDCT (C). For untreated mice, the simulated cellular responses include tumor volume and mRNA for TCR, IFNG, and TNFα. For immunized mice, the simulated cellular responses include total luciferase, active CD8+ T cells (CTLs) in the lymph node, active CTLs in blood, tumor volume, and mRNA for TCR, IFNG, and TNFα. Values of the PSRF less than 1.2 suggest convergence of the chains.

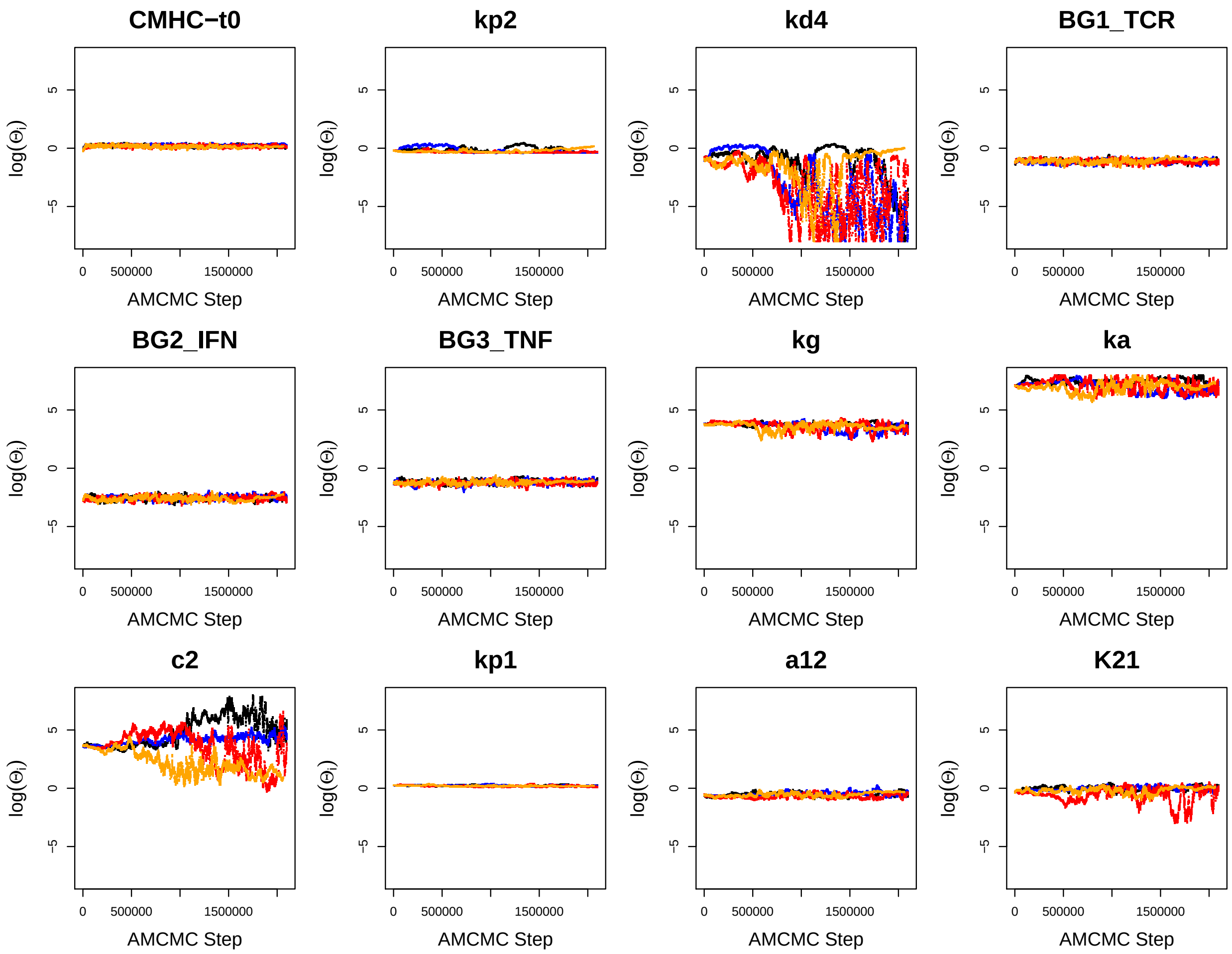

Figure S5. AMCMC summary plots for each of the model parameters associated with the V3 model. The trace of each of the model parameters is shown as a function of AMCMC step, where the parameter name is indicated above the panel. The traces for four parallel chains are shown in different colors: chain 1 (blue), chain 2 (black), chain 3 (red), and chain 4 (green). The three panels are the trace of the posterior likelihood ($P(Y|M, \theta)$) for each of the AMCMC chains with respect to untreated, rHuAd5-hgp100, and rHuAd5-hDCT conditions.

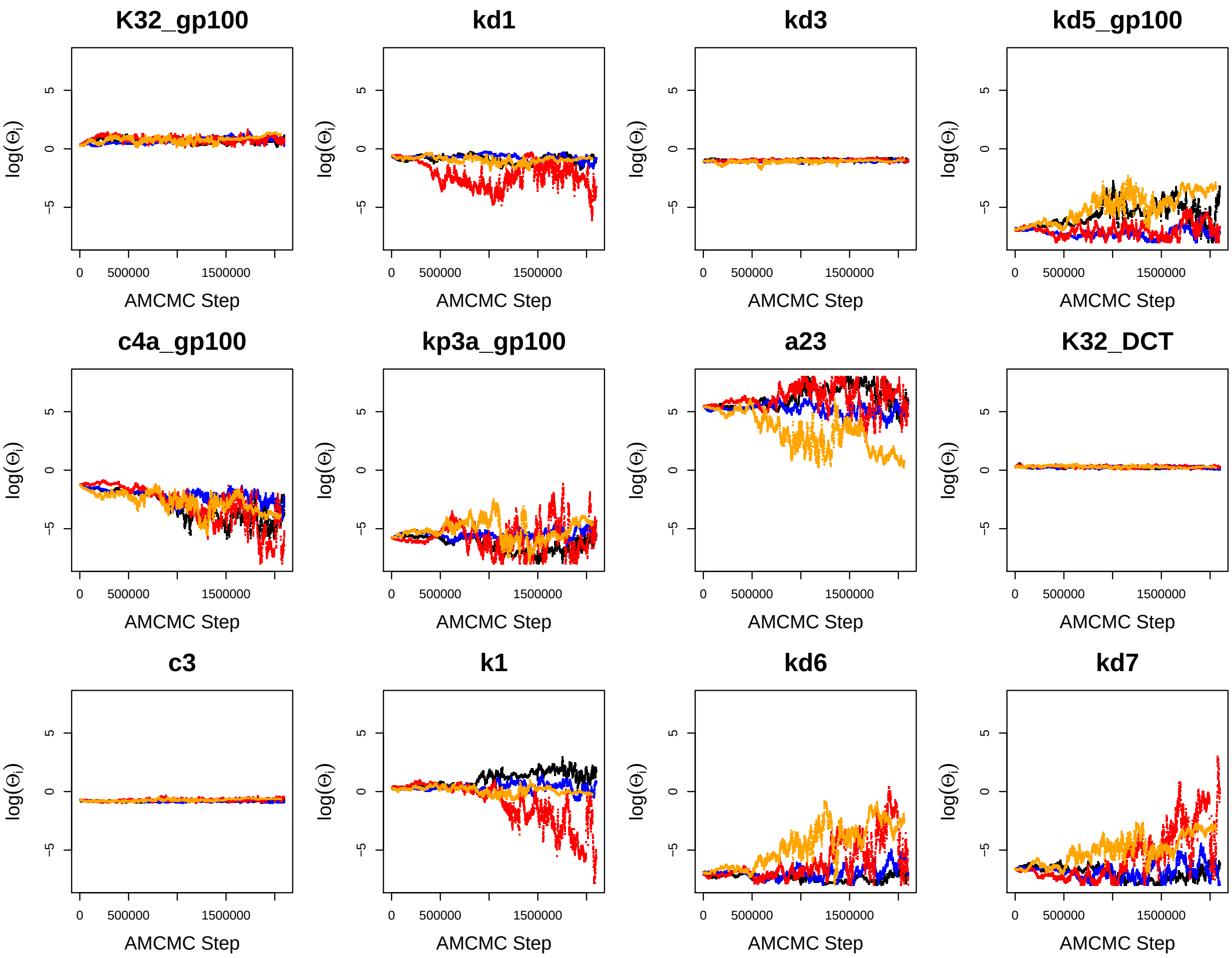

Figure S5 - continued.

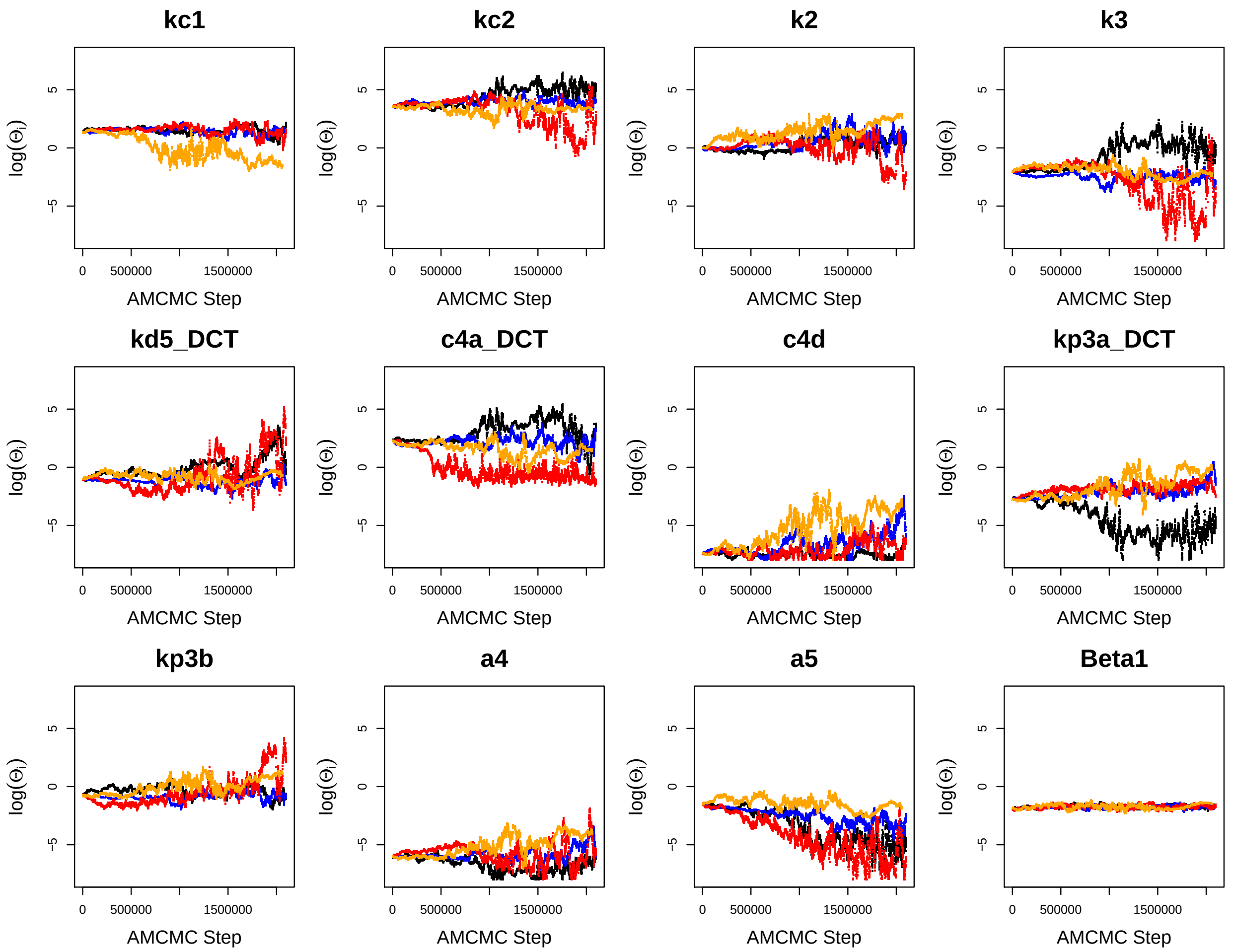

Figure S5 - continued.

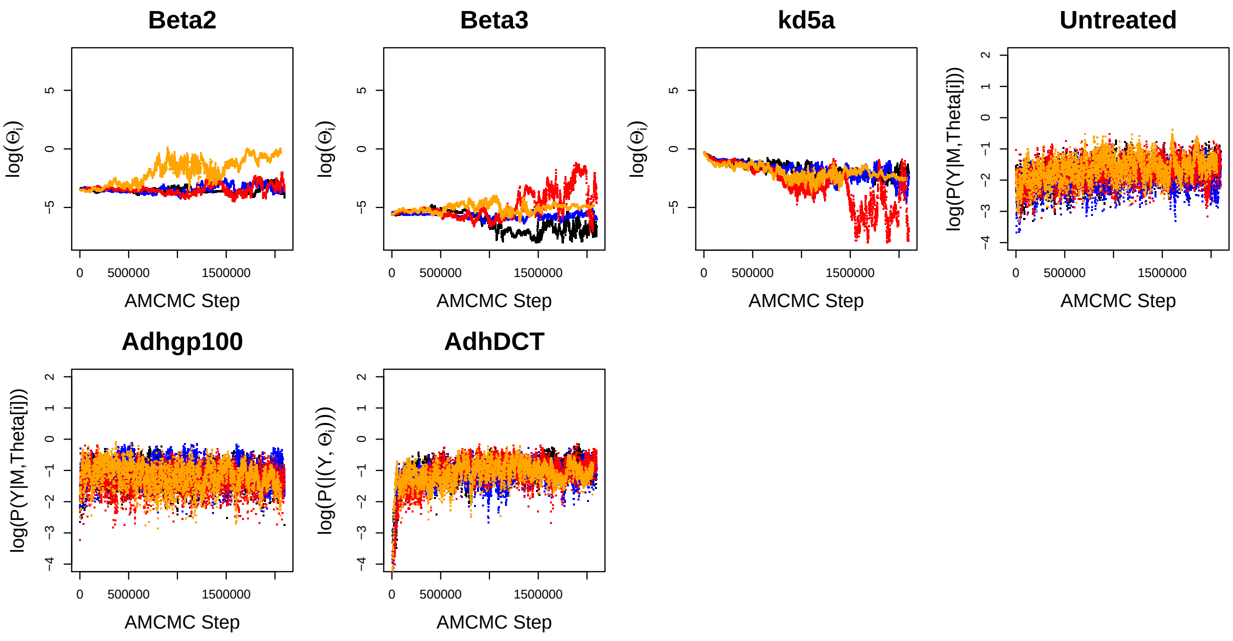

Figure S5 - continued.

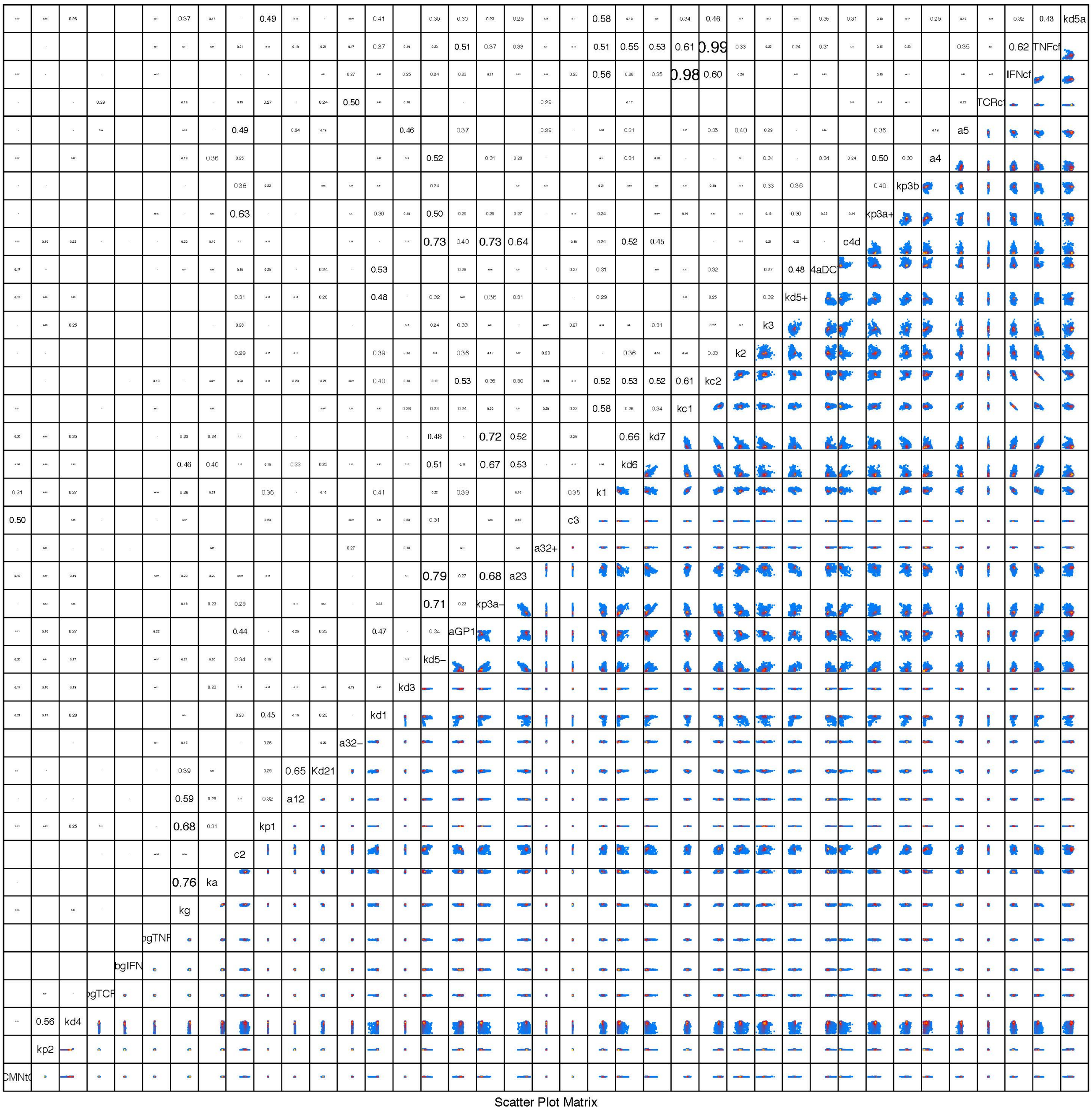

Figure S6. Pairwise comparison of posterior distribution in parameter values corresponding to the V3 model. Parameter names are given on the diagonal. Above the diagonal are the pairwise correlation coefficients of the parameters obtained from the four thinned Markov chains, where the font size is proportional to the value of the correlation coefficient. Pairwise projections of the marginalized probability density in $\log_{10}$ space are given below the diagonal. Coloring is based upon the estimated 2-D posterior density distributions using kernel density estimation. The axes for the scatter plots each spans from 10^{-8} to 10^{8}.

REFERENCES

[1]Abbas A., and A. Lichtman. 2003. *Cellular and Molecular Immunology*. Saunders Publishing, New York, 5th edition.

[2]Dominguez-Gerpe L.,and M. Rey-Mendez. 2001. Alterations induced by chronic stress in lymphocyte subsets of blood and primary and secondary immune organs of mice. *BMC Immunol.*, 2:7.

[3]Linderman, J. J., T. Riggs, M. Pande, M. Miller, S. Marino, and D. E. Kirschner. 2010. Characterizing the dynamics of CD4+ T cell priming within a lymph node. *J. Immunol.*, 184(6):2873–2885.
